# Transcriptomic profiling identifies ferroptosis and NF-κB signaling involved in α-dimorphecolic acid regulation of microglial inflammation

**DOI:** 10.1186/s12967-025-06296-7

**Published:** 2025-03-04

**Authors:** Xiao-Xi Zhu, Pei-Juan Wang, Shan Chao, Wei-Jia Tang, Long-You Zhao, Li-Mei Yu, Fan Yang

**Affiliations:** 1 Key Laboratory of Cell Engineering in Guizhou Province, Affiliated Hospital of Zunyi Medical University, Zunyi, China; 2 Department of Psychiatry, Nantong Fourth People’s Hospital, Nantong, China; 3 Research Center for Lin He Academician New Medicine, Institutes for Shanghai Pudong Decoding Life, Shanghai, China; 4 Lishui Key Laboratory of Brain Health and Severe Brain Disorders. Lishui Second People’s Hospital, Lishui, China; 5 Bio-X Institutes, Key Laboratory for the Genetics of Developmental and Neuropsychiatric Disorders (Ministry of Education), Shanghai Jiao Tong University, Shanghai, China

**Keywords:** Ferroptosis, NF-κB signaling, α-dimorphecolic acid, Microglial inflammation, Multiple sclerosis, Metabolomics, Transcriptomics

## Abstract

**Background:**

Microglia-evoked neuroinflammation contributes to neurodegenerative diseases such as multiple sclerosis (MS). Metabolic reprogramming, including changes in polyunsaturated fatty acids (PUFAs), plays a critical role in MS pathophysiology. Previous studies identified reduced plasma α-dimorphecolic acid (α-DIPA), a linoleic acid derivative, in MS patients. This study investigated the anti-inflammatory effects of α-DIPA on microglia and the underlying pathways.

**Methods:**

Lipopolysaccharide (LPS)-induced BV-2 microglial inflammation was used as an in vitro model. α-DIPA effects were assessed via ELISA for nitric oxide (NO) release, flow cytometry was used to examine cell proliferation, activation and polarization, and transcriptomic analysis was applied to identify key signaling pathways regulated by α-DIPA.

**Results:**

ELISA results showed that exogenous α-DIPA treatment significantly inhibited LPS-induced NO release from BV-2 cells in a concentration-dependent manner. Moreover, flow cytometry analysis suggested that 40 µM α-DIPA treatment significantly repressed LPS-induced BV-2 cell proliferation, activation, as well as M1 and M2 type polarization. Furthermore, transcriptome analysis revealed that exogenous α-DIPA extensively and drastically decreased the transcriptional level of numerous genes that are involved in the regulation of inflammatory responses, for instance, proinflammatory genes such as *Tnf* and *Ccl3* related to IL-17 and TNF-α signaling. In addition, we also observed that the expression of multiple genes in NF-κB signaling were also inhibited greatly by α-DIPA, such as *Nfkb2* and *Nfkbia*. Notably, α-DIPA robustly suppressed LPS-induced mRNA expression of abundant genes participating in the ferroptosis pathway, including *Acsl4*, *Slc7a11*, *Me1*, and *Hmox1*. Interestingly, the expressions of multiple ferroptosis-related genes were regulated specifically by α-DIPA but not LPS, such as *Acsl5*, *Acsl6*, *Alox5*, *Cars*, *Dpp3*, *Dpp10*, *Slc2a5*, and *Slc7a1*.

**Conclusion:**

α-DIPA inhibits microglial inflammation likely through regulating the pathways of the ferroptosis and NF-κB signaling. These results provided preliminary evidence for α-DIPA as a potential therapeutic candidate for neurodegenerative diseases like MS.

**Supplementary Information:**

The online version contains supplementary material available at 10.1186/s12967-025-06296-7.

## Introduction

Multiple sclerosis (MS) is a chronic inflammatory disease caused by demyelination of nerve axons that occurred in the central nervous system (CNS), which gives rise to CNS focal lesions, progressive neurodegeneration, cognitive impairment, paralysis, and even severe disability [1–3]. MS primarily affects young adults, particularly women compared to men [4]. MS affects approximately 2.8 million people worldwide [5], and imposes a considerable burden on individuals and healthcare systems, leading to substantial disability and reduced quality of life [6]. Current treatment strategies for MS focus on mitigating disease progression and managing symptoms through immunomodulatory and immunosuppressive therapies [7–9]. However, these treatments often have limited efficacy and are associated with significant side effects, posing challenges in long-term disease management. Furthermore, the complex and heterogeneous nature of MS pathophysiology complicates the identification of effective therapeutic targets, making it difficult to achieve complete remission [10, 11]. One of the critical pathological mechanisms in MS involves chronic inflammation mediated by neuroglial cells, including microglia, astrocytes, and oligodendrocytes [12]. Microglia, the resident immune cells within the CNS, play a pivotal role in initiating and sustaining inflammatory responses that is critical in neurodegenerative diseases like MS. Activated microglia release pro-inflammatory cytokines, chemokines, and reactive oxygen species, which contribute to the demyelination and neurodegeneration observed in MS. Usually, they play dual roles: protective by clearing protein aggregates and detrimental when overburdened, leading to impaired phagocytosis, neuroinflammation, and neurodegeneration [13–17]. Astrocytes exacerbates neuronal damage and disrupts the blood-brain barrier in CNS inflammation [18–23]. Oligodendrocytes, key to axon myelination, suffer from CNS inflammation, causing myelin loss and axonal dysfunction [24–27]. Together, these glial-mediated inflammatory processes are central to the pathogenesis of MS. Despite advancements in understanding the role of neuroglial cells in MS, there remains a lack of curative treatments targeting these cells. Early interventions aimed at modulating microglial inflammation hold promise as novel therapeutic strategies for MS.

PUFAs play a significant role in regulating microglial inflammation and MS pathology [28]. For example, linoleic acid (LA), one of ω-6 PUFA, has been shown to modulate immune responses and inflammation within the CNS [29, 30]. Previous studies have demonstrated that LA and its derivatives can influence the production of pro-inflammatory and anti-inflammatory mediators in microglial cells, thereby impacting the progression of neuroinflammatory diseases like MS [31–36]. The regulation of microglial activity by LA highlights the potential of dietary fatty acids as modulators of neuroinflammation and MS pathology. In particular, α-DIPA (also known as hydroxyoctadecaenoic acid, HODE), a secondary oxidative product of LA, which has been studied for its functional role in various disease contexts [37–42]. Concentrations of 9-(*S*)- and 13(*S*)-HODE have been found to vary significantly in disease states [43–46], suggesting their potential involvement in pathological events. Early study has indicated that HODEs can modulate macrophage-mediated inflammatory responses [47], implicating its potential role in regulation of inflammation. Recent study has demonstrated that chemically synthesized HODEs exhibits anti-inflammatory activities in vivo [48], further underscoring the function of HODEs in controlling inflammatory reactions. Although growing evidence suggested that α-DIPA may serve as therapeutic targets in the treatment of neuroinflammatory conditions, however, the pathophysiological role of α-DIPA in microglial inflammation, the key fundamental of MS pathology, remains largely unexplored. The current study aims to elucidate the role of α-DIPA in modulating microglial activation and inflammation through pathways such as ferroptosis and NF-κB signaling, offering insights into potential therapeutic strategies for MS.

In this study, we revealed that exogenous α-DIPA treatment significantly inhibited LPS-induced inflammatory events and regulates the expression of genes involved in ferroptosis and NF-κB signaling in microglial cells. Thus, we hypothesize that exogenous α-DIPA inhibits microglial inflammation through modulation of ferroptosis and NF-κB signaling, offering potential therapeutic relevance for MS. Besides, the inhibitory role of α-DIPA on microglia inflammation implying that reduced level of endogenous α-DIPA may be one of pathological factor eliciting microglial inflammation in CNS, as well as onset and progression of MS. Our in vitro findings suggested that α-DIPA exhibits potential anti-inflammatory effects that warrant further investigation as a possible therapeutic approach for MS management in the future.

## Methods and materials

### Subject recruitment and sample collection

Previously, 22 patients with MS and 21 matched healthy controls (HCs) were recruited, all HCs were strictly matched to MS patients based on age, gender, and body mass index (BMI). The demographic details (such as age, gender distribution, and BMI) of all subjects were shown in the Supplementary Table 1. Sample size of MS patients and HCs was determined using G*Power software. Written informed consent was obtained from each participant prior to the enrollment. The protocols of this study were reviewed and approved by the Ethics Committee of Lishui Second People’s Hospital (approval number 20171116-3), and the Ethics Committee of Affiliated Hospital of Zunyi Medical University (approval number KLL-2022-305). Detailed information about clinical diagnosis, sample collection, subject inclusion criteria and exclusion criteria, as well as medication status of MS patients, have been explained in detail in previous published articles [49, 50]. Peripheral blood samples were collected from all participants using EDTA-anticoagulant vacuum blood collection tubes. Subsequently, blood samples were centrifuged to separated plasma and stored at -80 °C for next study.

### LC-MS/MS-based metabolomic analysis

Liquid chromatography-mass spectrometry/mass spectrometry (LC-MS/MS)-based global untargeted metabolomics analysis approach was used to determine differentially abundant metabolites in MS-affected patients compared to HCs. All information about LC-MS/MS assay, including data acquisition, data processing, data normalization, data quality control, identification of isotope internal standards, identification of differentially abundant metabolites, bioinformatics analysis, and statistical analysis have been documented detailly in the previous published study [49]. The QC-RFSC algorithm in statTarget package (v.1.28.0) was used to correct batch effects.

### LPS-induced NO release assay

Mouse glioma cell line BV-2 (FengHui Biotech, Cat no. CL0056, Changsha, China) was recovered and cultured using DMEM/F12 cell culture medium (Gibco, Cat no. 11320033, USA), for 18 h in a 96-well plate, then was pretreated with 10, 20, 40, and 80 µM α-DIPA (GlpBio, Cat no. GC19460, USA) for 1 h, subsequently, BV-2 cell was further treated with 1.0 µg/mL LPS (Beyotime, Cat no. S1732, Shanghai, China) for another 24 h. Meanwhile, 100 µM of NG-monomethyl-L-arginine (L-NMMA) (Beyotime, Cat no. S0011, Shanghai, China), a total inhibitor of NO synthetase (NOS), was used as positive control. Each group had five biological replicates. After α-DIPA treatment, Enzyme-linked immunosorbent assay (ELISA) was performed to detect the level of NO released from BV-2 cells using NO detection Kit (Jiancheng Bioengineering Institute, Cat no. A012-1-2, Nanjing, China) according to manufacturer’s protocol. NO levels were normalized as a percentage change relative to untreated controls to account for baseline variability between experiments.

Biological replicates were randomly distributed across all treatment groups to ensure that each group consisted of samples from different individuals. Each replicate was independently prepared to maintain the integrity of the experimental conditions and to allow for the assessment of inter-individual variability. To determine the baseline level of NO release, we included a control group of untreated cells that were not exposed to LPS. All reagents used in this study, including α-DIPA, were sourced from reputable vendors and were subjected to rigorous validation for purity and stability before use. The purity of α-DIPA was confirmed to be ≥ 98% by high-performance liquid chromatography (HPLC) analysis, as provided by the manufacturer’s certificate of analysis. Additionally, the stability of α-DIPA was assessed under the storage conditions recommended by the vendor to ensure that the compound remained stable throughout the duration of the experiments. The validation process included a batch-to-batch variability test to ensure consistency in our experiments.

### Flow cytometry assay

BV-2 cell was recovered and cultured for 18 hr in a 6-well plate, then was pretreated with 40 µM α-DIPA for 1 hr, which was followed by further treatment with 1 µg/mL LPS for another 24 hr. Each group had three biological replicates. 5-bromo-2’-deoxyuridine (BrdU) (YEASEN Biotech, Cat no. 40204ES60, Shanghai, China) embedding experiment assay was performed to examined the proliferation status of BV-2 cells according to manufacturer’s protocol, flow cytometry assay was carried out to examined the activation status of BV-2 cells using CD68 marker, and examine the M1 and M2 type polarization status of BV-2 cells using CCR7, CD206, and CD163 markers, respectively. Briefly, 0.25% pancreatase was used to digest BV-2 cells. Antibodies of CD68 (BioLegend, Inc., Cat no. 137001, San Diego, CA, USA), CCR7 (BioLegend, Inc., Cat no. 120101, San Diego, CA, USA), CD206 (BioLegend, Inc., Cat no. 141713, San Diego, CA, USA), and CD163 (BioLegend, Inc., Cat no. 156702, San Diego, CA, USA) were diluted to 1:100 with 1× PBST solution that containing 1% BSA and 4’,6-diamidino-2-phenylindole (DAPI, 1:10 dilution), and incubated BV-2 cells for 15 min, 1× PBST solution was used to wash BV-2 cells for two times and resuspend cells with 500 µL 1× PBS solution before performing flow cytometry assay.

A total of three biological replicates were performed for each group under different experimental conditions. Fluorescence compensation controls were performed to ensure accurate flow cytometry data and to account for any spectral overlap between fluorophores. This process involved the use of single-stained controls for each fluorophore to create a compensation matrix. The matrix was then applied to the experimental data to adjust for any spillover of fluorescence signals into other channels. In our flow cytometry analysis, a systematic gating strategy was employed to define positive populations. Initially, a doublet discrimination gate was applied to exclude doublet events based on the height and area of the forward scatter (FSC) and side scatter (SSC) signals. Subsequently, a live/dead gate was used to exclude dead cells and cellular debris. For dead cell exclusion, we utilized DAPI staining, which allows for the discrimination of live from dead cells based on their nuclear permeability. DAPI-negative cells, indicating intact cell membranes and thus viable cells, were selected for further analysis. Following the exclusion of doublets and dead cells, fluorescence gates was applied to identify positive populations based on the staining for specific surface markers. In the interpretation of flow cytometry data, the efficiency of antibody binding and non-specific staining are potential sources of variability that could affect the accuracy of our results. To minimize these effects, we used highly specific antibodies that have been previously validated for their target antigens and performed appropriate isotype controls to account for non-specific binding.

### Transcriptome sequencing

#### Library preparation and high-through sequencing

Total RNAs were purified from BV-2 cells with number of 1 × 10^7^ by using TRNzol Universal total RNAs Purification Kit (TIANGEN, Cat no. DP424, China). 30 µL of nuclease-free ddH_2_O was used to dissolve total RNAs. The purity and concentration of total RNAs were examined using Nanodrop 2000c (ThermoFisher Scientific, USA) and 0.8% agarose gel electrophoresis. RNA integrity was assessed using the RNA Integrity Number (RIN) values, which were determined using an Agilent 2100 Bioanalyzer. Only samples with RIN values ≥ 7.0 were used for preparing library. Further, Equalbit RNA HS Assay Kit (Vazyme, Cat no. Equations 211-01, Nanjing, China) was used for quantifying RNAs concentration.

VAHTS Universal V10 RNA-Seq Library Prep Kit (Vazyme, Cat no. NR606-02, Nanjing, China) was used for preparing RNA library according to manufacturer’s protocol. 1× dsDNA (HS) Quantification Kit was used to determining the concentration of cDNA library using Qubit (ThermoFisher Scientific, USA), and the size of cDNA library was examined using Qsep400. High-through sequencing was conducted on an Illumina NovaSeq6000 platform with paired-end 150 bp approach. At least 6 Gb raw data was generated for each sample. The sequencing depth of 6 Gb per sample was chosen based on previous studies and our power analysis, which indicated that this depth provides sufficient coverage to detect low-abundance transcripts with a high level of confidence. This depth also allows for robust differential gene expression analysis and minimizes the impact of sequencing depth on the variability of the results. To minimize batch effects, we employed a randomized sample preparation strategy, ensuring that samples from different treatment groups were processed and sequenced in parallel.

#### Bioinformatic analysis

Raw data were filtered by fastp (v.0.21.0) to remove low quality reads and adapters, duplicate reads were removed and PCR artifacts were filtered out using the fastqc tool (v.0.11.9), to ensure the accuracy of our sequencing data. These preprocessing steps identifies and removes any adapter sequences, low-quality reads, and potential contaminants from the raw data. Filtered clean data was mapped to reference genome GRCh38 using Hisat2 (v.2.1.0) to generate Sam profile, which was subsequently converted into Bam profile using Samtools (v.1.9). FeatureCounts (v.2.0.3) method was used to quantifying messenger RNAs. StringTie (v1.3.4d) was used to annotate messenger RNAs. This annotation dataset, in “gene transfer format” (gtf), was obtained from ENSEMBL (release 94). The script provided by Stringtie (v.1.3.4d) was used to generate the expression matrix of counts instead of Fragments Per Kilobase of exon model per Million mapped reads (FPKM). Downstream statistical analysis was performed by R package (v.4.3.1) DESeq2 (v.2.14.1). The count data was normalized using default parameters. The fold change (FC) was adjusted by LfcShrink function of DESeq2. Finally, FC and difference significance were used to screen the differentially expressed genes (DEGs). mRNAs expression with Log_2_(FC) value greater than 1.0 or lower than − 1.0, and a *p* value lower than 0.05 were considered significantly up-regulated or down-regulated DEGs, respectively. Log_2_(FC) > 1.0 and *p* < 0.05 is a commonly accepted criterion in the field to indicate a biologically meaningful mRNA expression change with statistically significant. The removeBatchEffect() method in Limma package (v.3.58.1) was used to remove batch effects. -Log_10_ (*p*) values of DEGs over 350 were uniformly set to a maximum of 350 in plotting volcano figure. For alternative splicing analysis, we used Rmats software (v.4.1.0) and set the significance thresholds at a false discovery rate (FDR) of 0.05 to identify differential splicing events. New transcripts were predicted using Stringtie software (v.1.3.4d).

Gene ontology (GO) and Kyoto Encyclopedia of Genes and Genomes (KEGG) pathway enrichment analysis were performed by R package ClusterProfiler (v.3.16.1) [51]. We considered a *p*-value cutoff of 0.05 and a minimum gene count of 5 for a pathway to be significantly enriched, which helps to identify biologically relevant pathways that are significantly associated with our DEGs. The Benjamini-Hochberg (BH) procedure was applied to control the FDR in pathway analyses. Protein-protein interaction (PPI) analysis was carried out using the online tool Search Tool for Recurring Instances of Neighbor (STRING) (https://www.string-db.org/) [52] with default parameters. The network figure was created using Cytoscape (v.3.8.0) method. Receiver operating characteristic (ROC) curve analysis was conducted using pROC in R package [53]. Heatmap was plotted using the Pheatmap package in R (v.3.3.2). Volcano plots, box plots, bar plots, and ROC curve plot were generated using the ggplot2 in R package. Multivariate statistical analysis such as principal component analysis (PCA) was conducted using R package prcomp function. Supervised analyses including partial least squares-discriminant analysis (PLS-DA) and orthogonal partial least squares-discriminant analysis (OPLS-DA) was performed using mixOmics in R package [54]. Venn analysis was carried out using the online tool VENNY2.1 (https://bioinfogp.cnb.csic.es/tools/venny/).

### Statistical analysis

To analyze the data (such as NO release), we employed a series of appropriate statistical tests using GraphPad Prism software (San Diego, CA, USA). Initially, normality of the data was assessed using the Shapiro-Wilk test. Since our data passed the normality test, we proceeded with parametric statistical analyses. Specifically, we used one-way ANOVA followed by Tukey’s multiple comparisons post-hoc test to compare the mean NO levels across different treatment groups. Besides, for non-parametric tests, such as the comparison of gene expression data, the Mann-Whitney U test was used to compare two independent groups and the Kruskal-Wallis test for more than two groups. Post-hoc analyses with Dunn’s correction were performed to correct for multiple comparisons when necessary. For multiple comparisons, FDR correction was applied using the BH procedure, an adjusted *p* values were used to determine statistical significance, with a threshold of adjusted *p* < 0.05 considered significant. All statistical tests were two-tailed, and statistical significance was set at *p* < 0.05.

## Results

### Reduced level of α-DIPA significantly corelate to MS

**Fig. 1:**
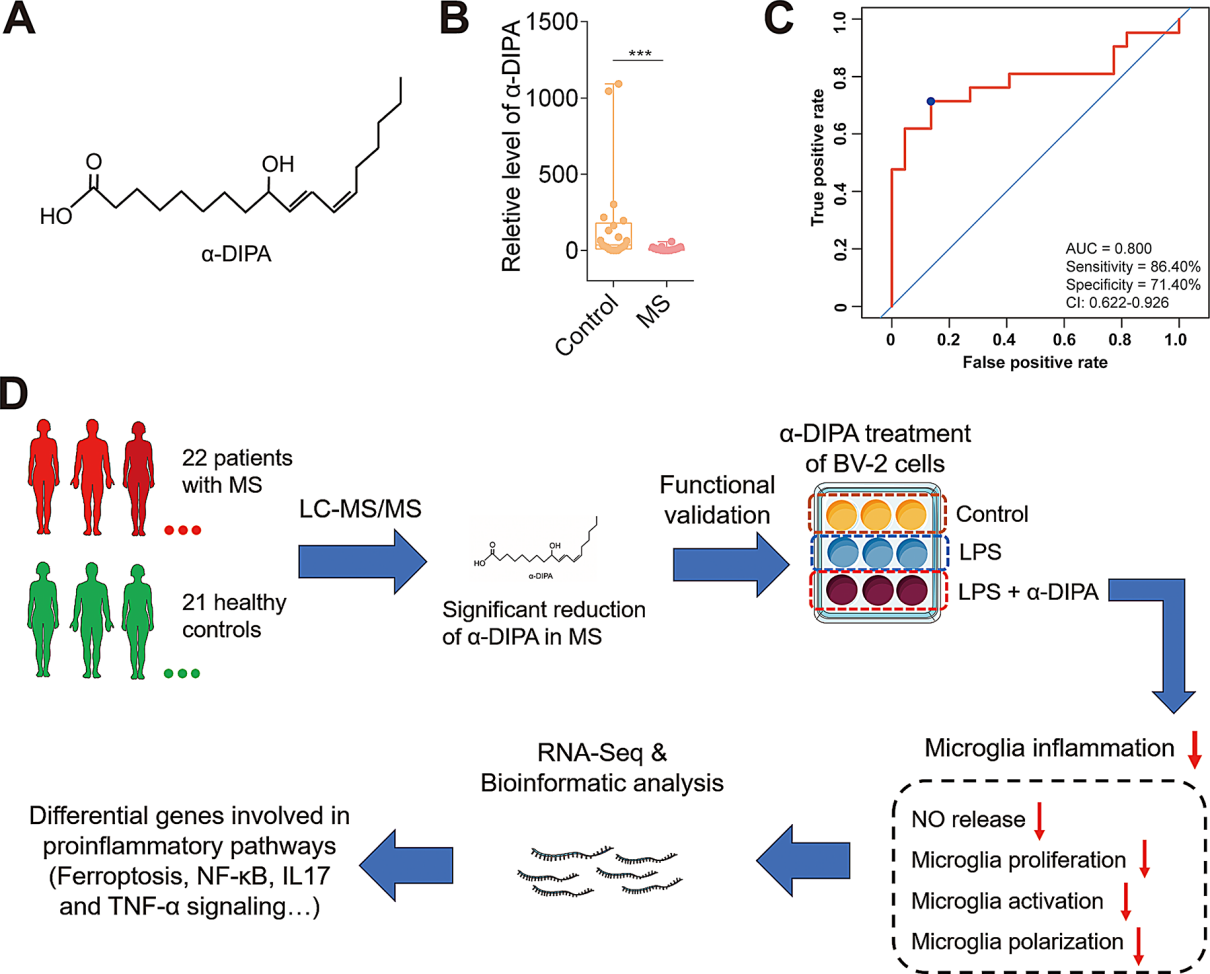
Significant association between α-DIPA deficiency and MS. (**A**) The chemical structure formula of α-DIPA molecule (C_18_H_32_O_3_). (**B**) Relative level of α-DIPA was significantly decreased in plasma of MS-affected patients (MS) compared to healthy controls (Control). Samples were compared using unpaired two-tailed *t*-test. *p* value was corrected using BH method. ***: adjusted *p* < 0.001. (**C**) Receiver operating characteristic (ROC) curve analysis was used to evaluate the potency of α-DIPA in predicting MS. 22 patients with MS and 21 healthy controls were used for ROC analysis. Area under curve (AUC): 0.800, sensitivity: 86.40%, specificity: 71.40%, 95% of confidence interval (CI): 0.622–0.926. (**D**) Schematic diagram represents the workflow of this study and its relationship with our previous study. Liquid chromatography-mass spectrometry/mass spectrometry (LC-MS/MS)-based metabolomics analysis of 22 patients with MS and 21 matched healthy controls was carried out in our previous study [49]

Previously, a total of 42 differentially abundant metabolites (DAMs) were identified in the plasma of MS-affected patients compared with HCs using LC-MS/MS-based global untargeted metabolomics approach, of which the plasma level of α-DIPA (Fig. 1A) was significantly reduced in patients with MS relative to those in HCs (adjusted *p* < 0.001) [49], with the greatest fold change (MS/HCs) of 0.06 among all DAMs (Fig. 1B). These results suggested a strong association between α-DIPA deficiency and MS, warranting further investigation into its potential etiological role. ROC analysis result showed that α-DIPA exhibited visible potency in predicting MS event with AUC of 0.800 (sensitivity = 86.40%, specificity = 71.40%, 95% of CI: 0.622–0.926) (Fig. 1C), which hinting plasma α-DIPA might be served as a potential assisting biomarker for MS diagnosis. Moreover, the reduction of α-DIPA was negatively corelated with the increased levels of IL-17 and TNF-α, while was positively corelated with the decreased levels of chemokines such as IL-8 and IL-12 [49], which implying that the change of α-DIPA level might be a latent metabolic regulator influencing inflammatory responses and even MS course. To investigate the biological role of α-DIPA in regulation of microglia inflammation that underlies MS etiopathology, and uncover underlying signaling pathways involved in this process, functional validation assays of α-DIPA combined with transcriptome sequencing were performed (Fig. 1D).

### Exogenous α-DIPA treatment was capable of inhibiting microglial inflammation

**Fig. 2:**
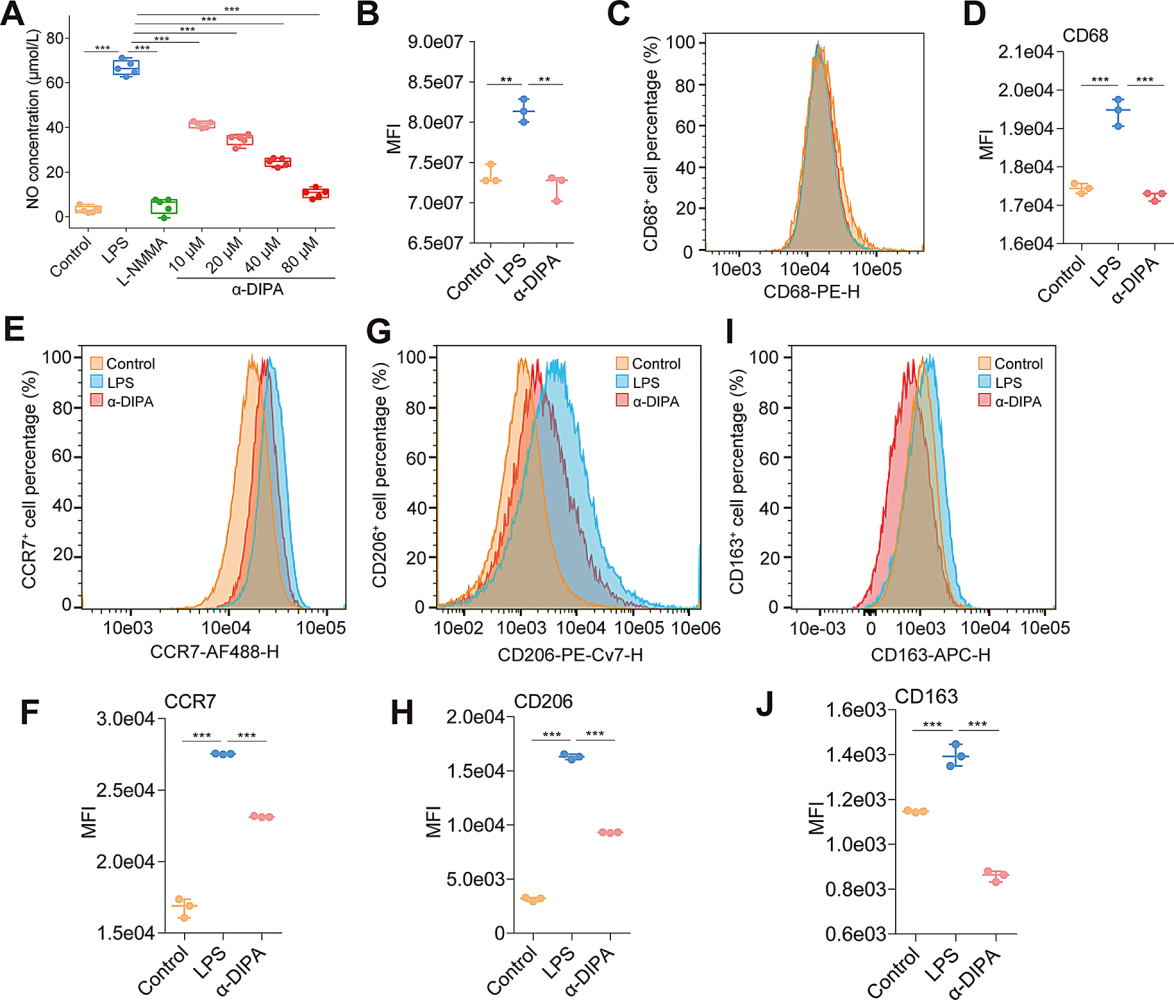
α-DIPA inhibits LPS-induced microglia inflammation. (**A**) α-DIPA inhibits lipopolysaccharide (LPS)-induced microglial NO release in a concentration-dependent manner. BV-2 cells treated with 1.0 µg/mL LPS, and various concentrations (10, 20, 40, and 80 µM) of α-DIPA. BV-2 cells treated without LPS was used as negative control to detect baseline of NO release, while BV-2 cells treated with 100 µM L-NMMA, a total inhibitor of NO synthetase, was used as positive control. Five biological replicates for each group. (**B-J**) Treatment of 40 µM α-DIPA for 1 h significantly inhibited LPS-induced BV-2 cell proliferation (**B**), activation (**C** and **D**), as well as M1 type (**E** and **F**) and M2 type (**G**-**J**) polarization. CD68 marker was used for staining activated BV-2 cells (**C** and **D**). CCR7 marker was used for staining BV-2 cells with M1 type polarization (**E** and **F**). While double staining of CD206 and CD163 markers were used for detecting BV-2 cells with M2 type polarization (**G**-**J**). BV-2 cells were treated with 1.0 µg/mL LPS for 24 h in (**B**-**J**). MFI in (**B**), (**D**), (**F**), (**H**), and (**J**) denotes mean fluorescence intensity. Three biological replicates for each group in (**B**-**J**). Samples were compared using one-way ANOVA statistical analysis and Tukey’s multiple comparisons post-hoc test, *: *p* < 0.05, **: *p* < 0.01, ***: *p* < 0.001

To determine whether α-DIPA play a biological role in regulating microglia-mediated inflammatory reactions, α-DIPA intervention of LPS-induced NO release assay was performed. ELISA results showed that 1.0 µg/mL LPS treatment for 24 h significantly increased NO release in BV-2 cells (*p* < 0.001), whereas the application of NOS inhibitor of NG-monomethyl-L-arginine (L-NMMA) completely blocked LPS-induced NO release (Fig. 2A). Similarly, the addition of exogenous α-DIPA significantly inhibited LPS-elicited NO release in a concentration-dependent manner (*p* < 0.001) (Fig. 2A). 40 µM and 80 µM of α-DIPA treatment for 1 h significantly reduced NO level to approximately 37% and 16% compared to that of LPS induction (*p* < 0.001), respectively (Fig. 2A). Besides, the inhibitory effects of α-DIPA on NO release were compared with those of L-NMMA, a well-established nitric oxide synthase inhibitor. Specifically, we calculated the percentage inhibition of NO release for both α-DIPA and L-NMMA treatments relative to LPS-induced controls. The magnitude of inhibition by 80 µM α-DIPA was found to be comparable to that of L-NMMA (Fig. 2A), indicating a significant suppressive effect on NO production.

To further determine whether α-DIPA was able to regulate LPS-induced microglia cells proliferation, activation, as well as M1 and M2 type polarization, BrdU staining and fluorescence-activated cell sorting (FACS) assay was carried out. BrdU embedding experiment result showed that 40 µM α-DIPA treatment for 1 h significantly repressed LPS-induced BV-2 cells proliferation (*p* < 0.001) (Fig. 2B). In addition, FACS assay results suggested that 40 µM of α-DIPA treatment significantly suppressed LPS-stimulated BV-2 cells activation (CD68^+^) (*p* < 0.001) (Fig. 2C and D). Furthermore, 40 µM of α-DIPA treatment for 1 h not only significantly inhibited LPS-induced M1 (CCR7^+^) type polarization of BV-2 cells (*p* < 0.001) (Fig. 2E and F), but it also greatly repressed M2 (CD206^+^ and CD163^+^) type polarization of BV-2 cells (*p* < 0.001) (Fig. 2G-J). These results suggested that exogenous α-DIPA play a robustly inhibitory role in microglia inflammation, and perhaps endogenous α-DIPA might play a functional role in inhibiting microglia-mediated inflammatory reaction in CNS.

### Transcriptomic dissection of the underlying signaling pathways participating in α-DIPA suppression of microglial inflammation

**Fig. 3:**
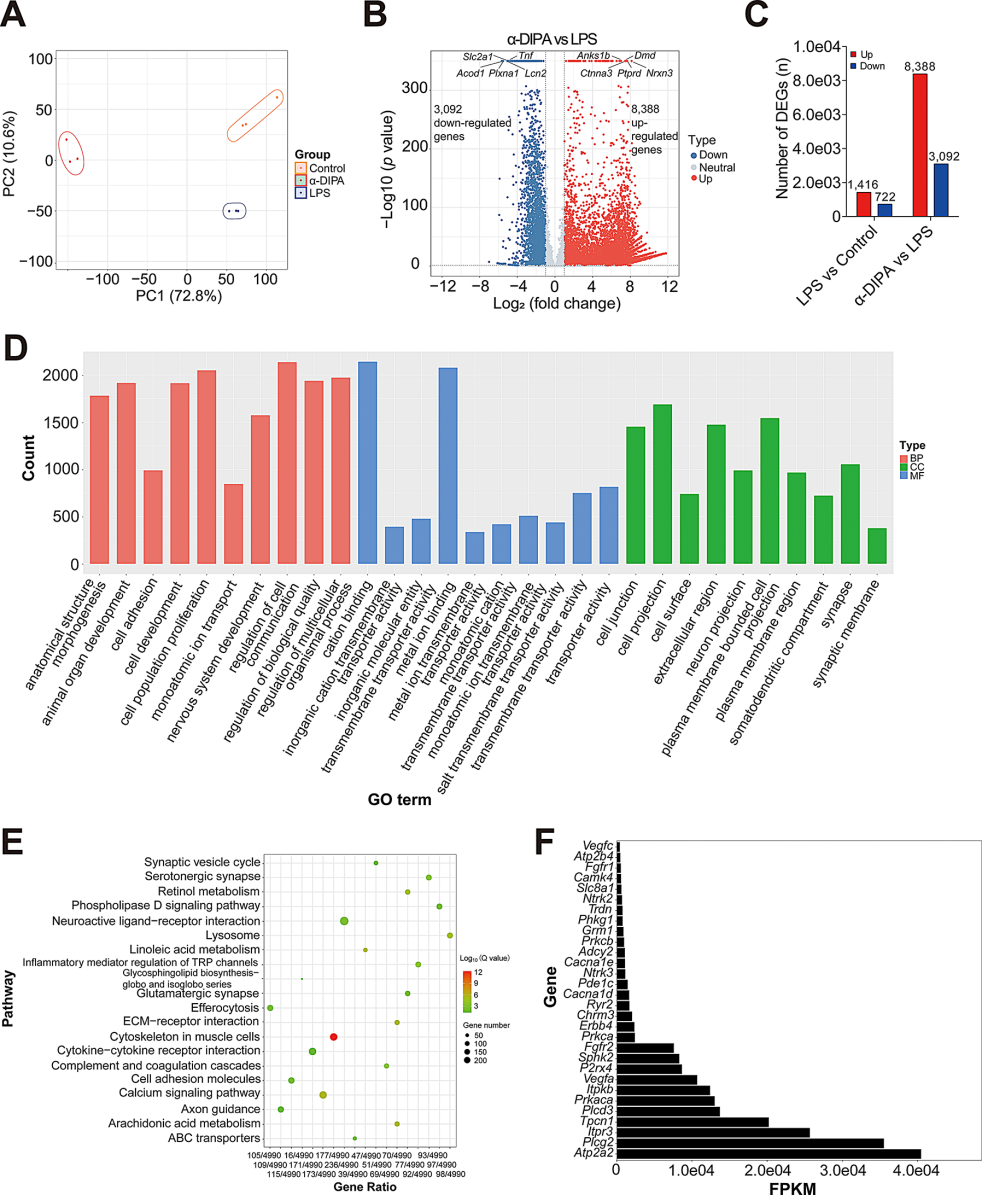
Transcriptomic analysis reveals α-DIPA-regulated genes and their enriched signaling pathways. (**A**) PCA analysis showed samples in the three groups of control, LPS, and α-DIPA, separated from each other. Three biological replicates were used in each group. (**B**) Volcano plot showed 11,480 differential genes regulated by α-DIPA, including 8,388 up-regulated (such as *Nrxn3*) and 3,092 down-regulated genes (such as *Tnf*). Log_2_(FC) > 1.0 or Log_2_(FC) < -1.0, and *p* < 0.05 was used to identify up-regulated or down-regulated genes, respectively. (**C**) Bar plot represented the number of up-regulated and down-regulated differentially expressed genes (DEGs) regulated by LPS and α-DIPA, respectively. (**D**) and (**E**) Gene ontology (GO) and Kyoto Encyclopedia of Genes and Genomes (KEGG) pathway enrichment analysis result of 11,480 DEGs regulated by α-DIPA. BP: biological process; CC: cellular compartment; MF: molecular function. (**F**) Top30 of most significant genes involved in calcium signaling pathway. FPKM: Fragments Per Kilobase of exon model per Million mapped reads

To further investigate the underlying signaling pathways involved in α-DIPA-inhibiting inflammatory reactions in microglia cells, transcriptome sequencing assay was conducted. Briefly, BV-2 cells were divided into three groups, including the group of control (BV-2 cells without treatment), LPS (BV-2 cells treated with 1.0 µg/mL LPS for 24 h), and α-DIPA (BV-2 cells pretreated with 40 µM α-DIPA for 1 h before LPS treatment). PCA analysis results showed that the three biological replicates exhibit grouping patterns based on different treatment (Fig. 3A). PCA plot revealed distinct clustering patterns that correspond to specific treatment groups. These groupings reflect the treatment-specific effects on the transcriptomic profiles of the cells. Notably, the separation of clusters along the first principal component (PC1) indicates a significant shift in the transcriptome due to α-DIPA treatment, suggesting a substantial impact on the underlying biological pathways. The distinct grouping of treated *versus* untreated samples underscores the robustness of the treatment effect and its discernible influence on the cellular transcriptome. The comparative group of LPS *versus* control and α-DIPA *versus* LPS was designated as C1 and C2, respectively. A total of 2,138 differentially expressed genes (DEGs), including 1,416 up-regulated [Log_2_(FC) > 1.0, and *p* < 0.05] and 722 down-regulated [Log_2_(FC) < -1.0, and *p* < 0.05] genes, were identified in the comparative group of C1 (Supplementary Fig. 1). And, a total of 11,480 DEGs, including 8,388 up-regulated [Log_2_(FC) > 1.0, and *p* < 0.05] and 3,092 down-regulated [Log_2_(FC) < -1.0, and *p* < 0.05] genes, were identified in the comparative group of C2 (Fig. 3B and C), which suggesting α-DIPA extensively regulate transcriptomic network participating inflammation process.

GO enrichment analysis results showed that α-DIPA-regulated DEGs primarily involved in the biological processes of anatomical structure morphogenesis, animal organ development, cell adhesion, cell development, cell population proliferation, monoatomic ion transport, nervous system development, regulation of cell communication, regulation of biological quality, and regulation of multicellular organismal process (Fig. 3D). Moreover, the molecular function of 11,480 DEGs was mainly cation binding, transporter activity, inorganic cation transmembrane transporter activity, inorganic molecular entity transmembrane transporter activity, metal ion binding, metal ion and salt transmembrane transporter activity, monoatomic cation and ion transmembrane transporter activity (Fig. 3D). Furthermore, the 11,480 DEGs-encoding proteins mainly localize in cell junction, cell projection, cell surface, extracellular region, neuron projection, plasma membrane region, somatodendritic compartment, synapse, and synaptic membrane (Fig. 3D). KEGG pathway enrichment analysis results showed that α-DIPA-regulated DEGs primarily participated in multiple signaling pathways, including synaptic vesicle cycle, serotonergic synapse, neuroactive ligand-receptor interaction, linoleic acid metabolism, inflammatory mediator regulation of TRP channels, glutamatergic synapse, efferocytosis, ECM-receptor interaction, cytokine-cytokine receptor interaction, cell adhesion molecules, calcium signaling pathway, axon guidance, arachidonic acid metabolism, as well as ABC transporters (Fig. 3E). Interestingly, we found majority of DEGs involved in cation binding, ion transport and calcium signaling pathway, such as top-ranking genes *Atp2a2*, *Plcg2*, *Itpr3*, *Tpcn1*, *Plcd3*, *Prkaca*, *Itpkb*, *Vegfa*, *P2rx4*, *Sphk2*, and *Fgfr2* (Fig. 3F).

**Fig. 4:**
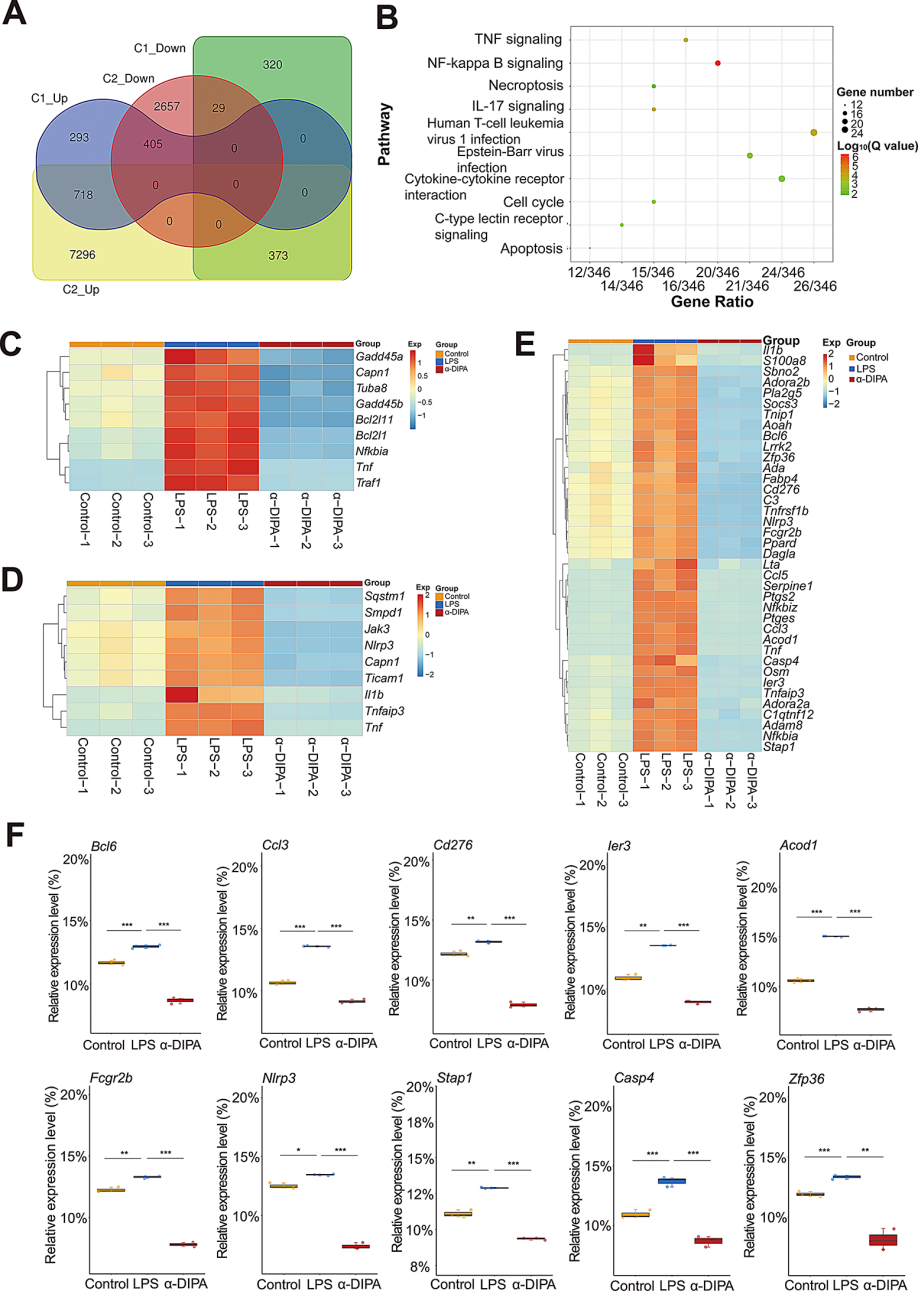
α-DIPA inhibits LPS-induced proinflammatory genes expression and their participating pathways. (**A**) Venn analysis showed 778 overlapping genes shared by comparative groups of C1 (LPS *versus* control) and C2 (α-DIPA *versus* LPS), including 405 overlapping genes shared by up-regulated DEGs of C1 and down-regulated DEGs of C2, and 373 overlapping genes shared by down-regulated DEGs of C1 and up-regulated DEGs of C2. (**B**) KEGG pathway enrichment analysis showed the common 778 genes enriched in proinflammatory pathways, such as NF-κB, IL-17 and TNF signaling. (**C**) and (**D**) Heatmap represented α-DIPA inhibits the mRNA expression levels of LPS-induced genes involved in apoptosis (**C**) and necroptosis (**D**), which may attenuate inflammatory cascade induced by abundant proinflammatory factors released from cell death. (**E**) and (**F**) Heatmap and box plot showed α-DIPA treatment significantly suppress the transcriptional levels of LPS-induced genes involved in regulation of inflammatory responses, including *Bcl6*, *Ccl3*, *Cd276*, *Ier3*, *Acod1*, *Fcgr2b*, *Nlrp3*, *Stap1*, *Casp4*, and *Zfp36*. Three biological replicates for each group in (**F**). Samples were compared using Kruskal-Wallis test. Post-hoc analyses with Dunn’s correction were performed. *: *p* < 0.05, **: *p* < 0.01, ***: *p* < 0.001. Exp in (**C**-**E**) means expression. Red and blue represents up- and down-regulation, respectively

To identify LPS-modulated DEGs and signaling pathways that also regulated inversely by α-DIPA in microglia, we analyzed the common DEGs between C1 and C2 group. Venn analysis results showed that 405 overlapping genes were shared by down-regulated DEGs in C2 and up-regulated DEGs in C1, and 373 overlapping genes were shared by up-regulated DEGs in C2 and down-regulated DEGs in C1 (Fig. 4A). KEGG pathway enrichment analysis showed the 778 DEGs primarily participated in NF-κB signaling, necroptosis, apoptosis, TNF-α signaling, IL-17 signaling, cytokine-cytokine receptor interaction, and C-type lectin receptor signaling (Fig. 4B). In particular, we observed α-DIPA robustly suppressed the expression level of multiple genes participated in apoptosis and necroptosis (Fig. 4C and D), which may attenuate inflammatory cascade induced by abundant proinflammatory factors released from cell death. Notably, α-DIPA treatment significantly repressed the mRNA expression level of LPS-induced genes involved in regulation of inflammatory responses (*p* < 0.01), such as *Bcl6*, *Ccl3* (i.e. *Mip-1α*), *Cd276*, *Ier3*, *Acod1*, *Fcgr2b*, *Nlrp3*, *Stap1*, *Casp4*, and *Zfp36* (Fig. 4E and F).

### α-DIPA inhibit the expression of proinflammatory genes involved in IL-17 and TNF-α signaling

**Fig. 5:**
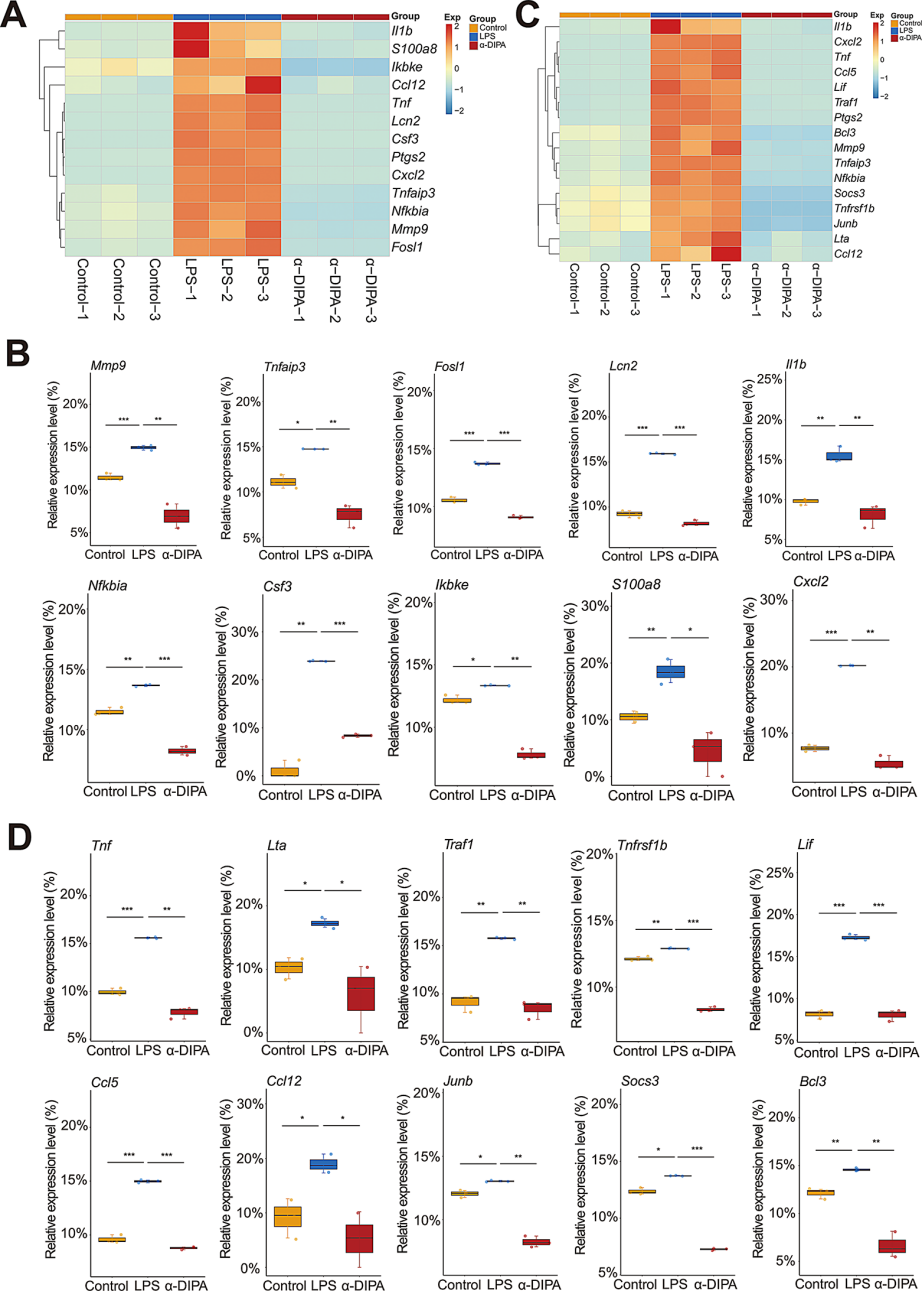
α-DIPA significantly repress expression levels of proinflammatory genes involved in IL-17 and TNF-α signaling. (**A**) and (**B**) Heatmap and box plot showed that the application of α-DIPA significantly decreased mRNA levels of LPS-induced genes involved in IL-17 signaling (**A**), including *Mmp9*, *Tnfaip3*, *Fosl1*, *Lcn2*, *Il1b*, *Nfkbia*, *Csf3*, *Ikbke*, *S100a8*, and *Cxcl2* (**B**). (**C**) and (**D**) Heatmap and box plot showed that α-DIPA treatment significantly reduced expression levels of LPS-induced genes involved in TNF-α signaling (**C**), including *Tnf*, *Lta*, *Traf1*, *Tnfrsf1b*, *Lif*, *Ccl5*, *Ccl12*, *Junb*, *Socs3*, and *Bcl3* (**D**). Three biological replicates for each group in (**A**-**D**). Samples were compared using Kruskal-Wallis test. Post-hoc analyses with Dunn’s correction were performed. *: *p* < 0.05, **: *p* < 0.01, ***: *p* < 0.001. Exp in (**A**) and (**C**) means expression. Red and blue represents up- and down-regulation, respectively

Transcriptome sequencing results showed that 1.0 µg/mL of LPS treatment greatly up-regulated the transcript levels of numerous pro-inflammatory genes involved in IL-17 signaling (Fig. 5A), including *Mmp9*, *Tnfaip3*, *Fosl1*, *Lcn2*, *Il1b*, *Nfkbia*, *Csf3*, *Ikbke*, *S100a8*, and *Cxcl2* (Fig. 5B). Whereas, intervention of 40 µM α-DIPA significantly down-regulated the expression level of LPS-induced genes (*p* < 0.05) (Fig. 5A and B). Also, α-DIPA treatment significantly suppressed the expression of LPS-induced inflammatory genes involved in TNF-α signaling (*p* < 0.05), including *Tnf*, *Lta*, *Traf1*, *Tnfrsf1b*, *Lif*, *Ccl5* (i.e. *Rantes*), *Ccl12*, *Junb*, *Socs3*, and *Bcl3* (Fig. 5C and D). Interestingly, previous study demonstrated that the circulating levels of TNF-α and IL-17 were significantly increased in MS-affected patients compared to HCs [49], suggesting the significant reduction of plasma α-DIPA level might be one of causal factors evoking activation of IL-17 and TNF-α signaling in patients with MS.

### α-DIPA repress microglia inflammation potentially through the ferroptosis and NF-κB signaling

**Fig. 6:**
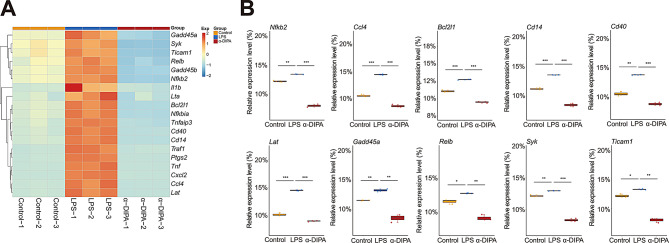
α-DIPA greatly reduces expression levels of proinflammatory genes in NF-κB signaling. (**A**) and (**B**) Heatmap and box plot represented that α-DIPA treatment largely decreased mRNA levels of LPS-induced genes involved in NF-κB signaling (**A**), including *Nfkb2*, *Ccl4*, *Bcl2l1*, *Cd14*, *Cd40*, *Lat*, *Gadd45a*, *Relb*, *Syk*, and *Ticam1* (**B**). Three biological replicates for each group. Samples were compared using Kruskal-Wallis test. Post-hoc analyses with Dunn’s correction were performed. *: *p* < 0.05, **: *p* < 0.01, ***: *p* < 0.001. Exp in (**A**) denotes expression. Red and blue represents up- and down-regulation, respectively

To further explore which signaling pathway likely mediate α-DIPA-regulating anti-inflammatory response, we further dissect the 778 common DEGs and its participating pathways. KEGG pathway enrichment analysis result showed that the 778 DEGs significantly enriched in NF-κB signaling pathway (Fig. 4B). We observed treatment of 40 µM α-DIPA significantly inhibited the expression level of LPS-induced pro-inflammatory genes that participated in NF-κB signaling (*p* < 0.01), such as *Nfkb2*, *Ccl4* (i.e. *Mip-1β*), *Bcl2l1*, *Cd14*, *Cd40*, *Lat*, *Gadd45a*, *Relb*, *Syk*, and *Ticam 1* (Fig. 6A and B), which implying NF-κB signaling might engaged in α-DIPA inhibition of inflammatory response. GO and KEGG pathway analyses have identified several DEGs that play critical roles in inflammation. For instance, within the NF-κB signaling pathway, we observed significant downregulation of DEGs such as *Tnf* and *Il1b*, which are key pro-inflammatory cytokines known to drive neuroinflammation in MS. The suppression of these genes by α-DIPA treatment suggests a direct anti-inflammatory effect on microglial inflammation.

**Fig. 7:**
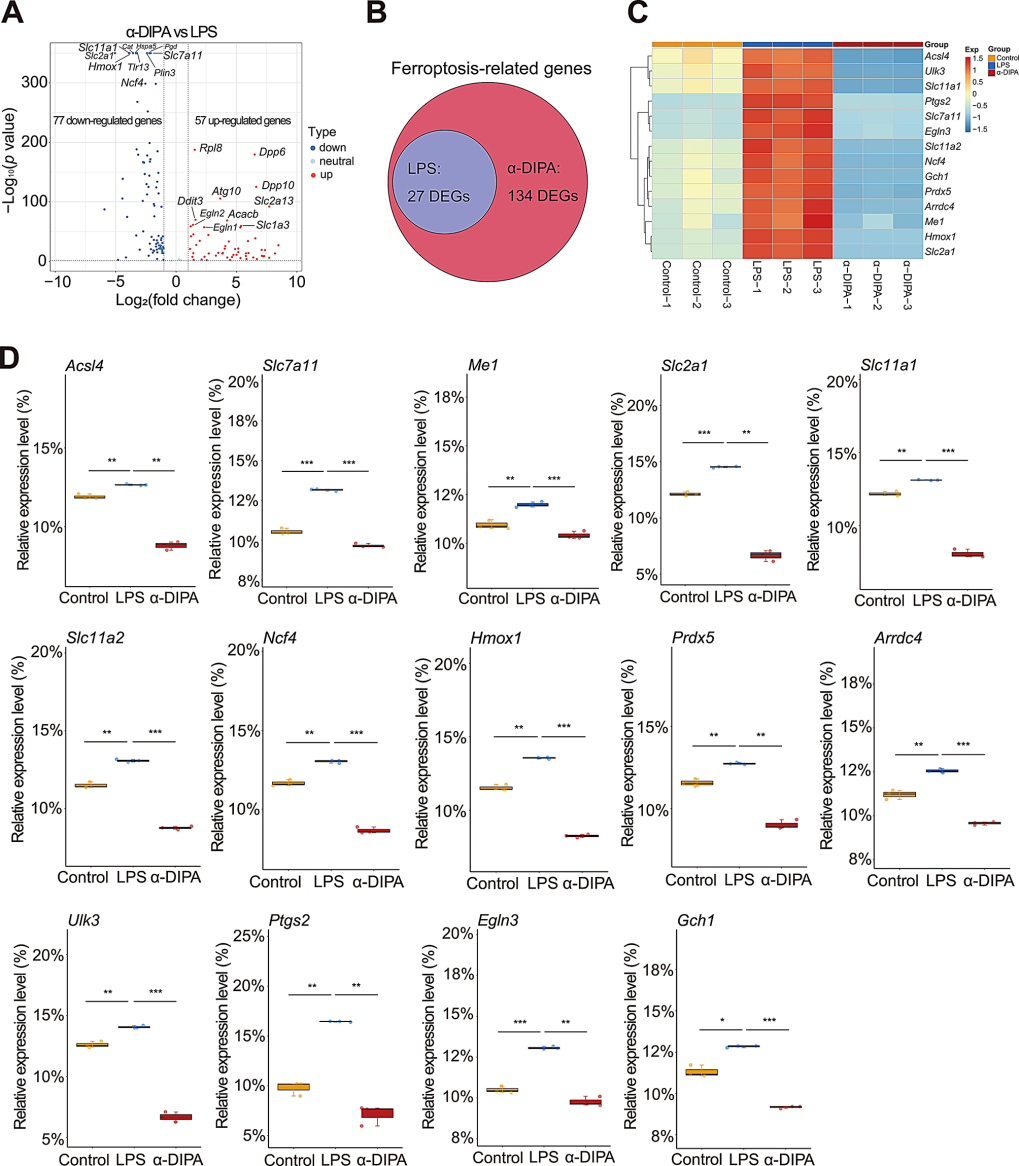
α-DIPA robustly reduces the expression of ferroptotic genes. (**A**) Volcano plot showed that α-DIPA treatment significantly up-regulated and down-regulated the expression levels of 57 and 77 ferroptotic genes, respectively, such as *Hmox1*, *Slc7a11*, *Dpp6*, and *Rpl8*. Log_2_(FC) > 1.0 or Log_2_(FC) < -1.0, and *p* < 0.05 was used to identify up- or down-regulated genes, respectively. (**B**) Venn plot represented 134 ferroptosis-related genes regulated by α-DIPA, of which 27 genes were also regulated by LPS. (**C**) and (**D**) Heatmap and box plot showed that α-DIPA treatment significantly inhibited the expression levels of LPS-induced ferroptotic genes (**C**), including *Acsl4*, *Me1*, *Slc7a11*, *Slc2a1*, *Slc11a1*, *Slc11a2*, *Ncf4*, *Hmox1*, *Prdx5*, *Arrdc4*, *Ulk3*, *Ptgs2*, *Egln3*, and *Gch1* (**D**), which potentially alleviate inflammatory reactions stimulated by ferroptosis and cell stress. Three biological replicates for each group in (**A**), (**C**), and (**D**). Samples were compared using Kruskal-Wallis test. Post-hoc analyses with Dunn’s correction were performed. *: *p* < 0.05, **: *p* < 0.01, ***: *p* < 0.001. Exp in (**C**) denotes expression. Red and blue represents up- and down-regulation, respectively

**Fig. 8:**
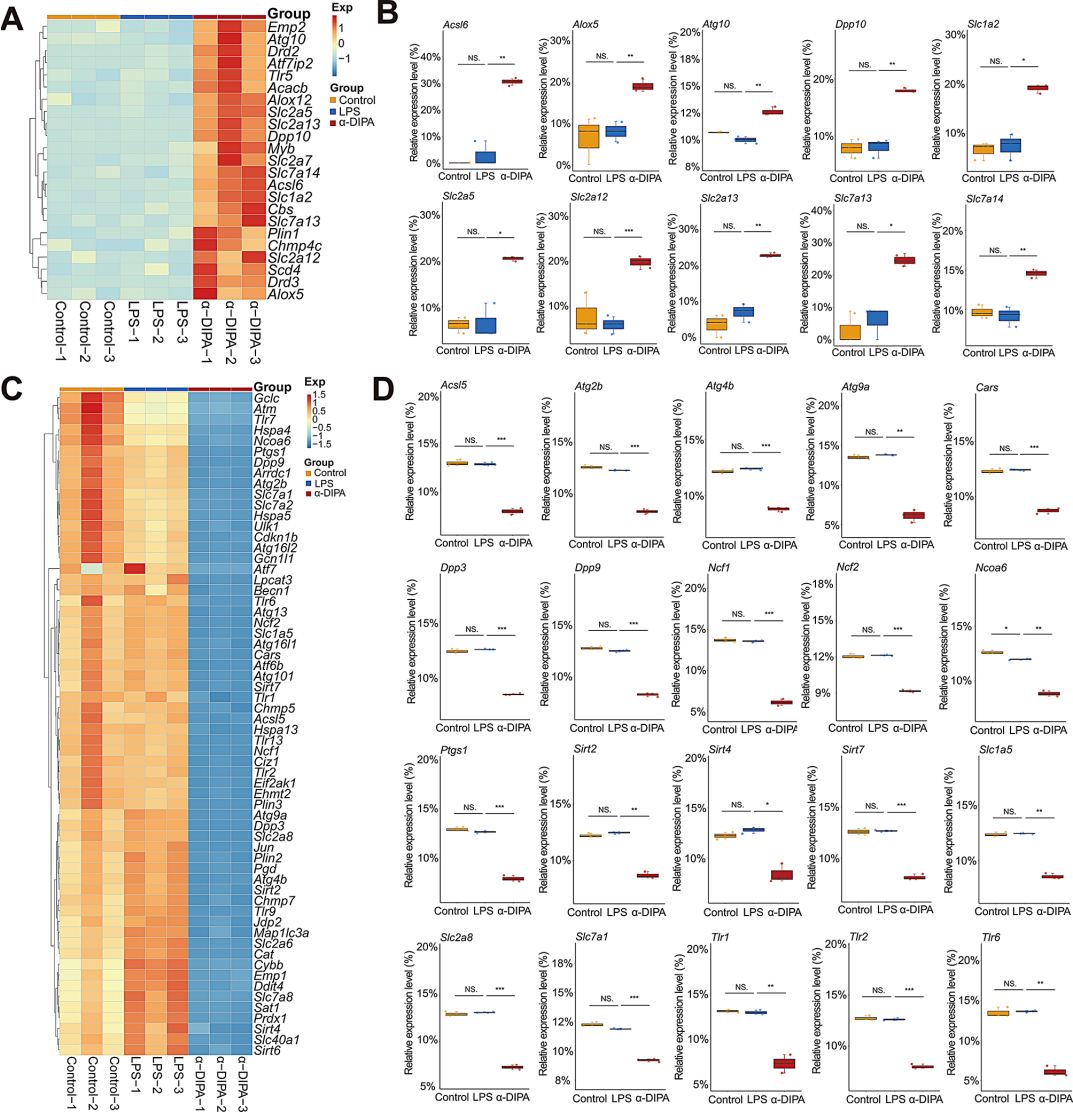
α-DIPA specifically regulates the expression level of ferroptosis-related genes. (**A**) and (**B**) Heatmap and box plot showed that α-DIPA but not LPS specifically increases the transcript levels of ferroptosis-related genes (**A**), including *Acsl6*, *Alox5*, *Atg10*, *Dpp10*, *Slc1a2*, *Slc2a5*, *Slc2a12*, *Slc2a13*, *Slc7a13*, and *Slc7a14* (**B**). (**C**) and (**D**) Heatmap and box plot represented that α-DIPA but not LPS specifically decreases the transcript levels of ferroptosis-related genes (**C**), including *Acsl5*, *Atg2b*, *Atg4b*, *Atg9a*, *Cars*, *Dpp3*, *Dpp9*, *Ncf1*, *Ncf2*, *Ncoa6*, *Ptgs1*, *Sirt2*, *Sirt4*, *Sirt7*, *Slc1a5*, *Slc2a8*, *Slc7a1*, *Tlr1*, *Tlr2*, and *Tlr6* (**D**). Three biological replicates for each group in (**A**-**D**). Samples were compared using Kruskal-Wallis test. Post-hoc analyses with Dunn’s correction were performed. *: *p* < 0.05, **: *p* < 0.01, ***: *p* < 0.001. NS: not significant. Exp in (**A**) and (**C**) means expression. Red and blue represents up- and down-regulation, respectively

Intriguingly, we also observed numerous DEGs enriched in programmed cell death process such as apoptosis and necroptosis (Fig. 4B-D). Furthermore, volcano plot showed α-DIPA pretreatment significantly regulate the expression level of 134 ferroptosis-related genes, including 57 up-regulated and 77 down-regulated DEGs, such as *Slc2a13*, *Dpp6*, *Dpp10*, *Rpl8*, as well as *Slc2a1*, *Hmox1*, and *Slc11a1* (Fig. 7A). By contrast, LPS treatment only changed the expression level of 27 ferroptotic genes (Fig. 7B), for instance, up-regulate genes *Ptgs2*, *Slc7a11*, *Hmox1*, *Slc2a1*, and down-regulated genes *Mybl2*, *Ncoa4*, and *Acsl3*, and *Cars2* (Supplementary Fig. 2). Moreover, α-DIPA treatment significantly reduced the transcription level of multiple ferroptosis-related genes that induced by LPS (*p* < 0.01), such as *Acsl4*, *Me1*, *Slc7a11*, *Slc2a1*, *Slc11a1*, *Slc11a2*, *Ncf4*, *Hmox1*, *Prdx5*, *Arrdc4*, *Ulk3*, *Ptgs2*, *Egln3*, and *Gch1* (Fig. 7C and D). Interestingly, we also observed a specific expression pattern of ferroptosis-related genes that regulated only by α-DIPA but not LPS. α-DIPA treatment significantly up-regulated the expression level of numerous ferroptotic genes (*p* < 0.05), such as *Acsl6*, *Alox5*, *Atg10*, *Dpp10*, *Slc1a2*, *Scl2a5*, *Slc2a12*, *Slc2a13*, *Slc7a13*, and *Slc7a14* (Fig. 8A and B), also, α-DIPA treatment greatly down-regulated the expression level of multiple ferroptotic genes, such as *Acsl5*, *Atg2b*, *Atg4b*, *Atg9a*, *Cars*, *Dpp3*, *Dpp9*, *Ncf1*, *Ncf2*, *Ncoa6*, *Ptgs1*, *Sirt2*, *Sirt4*, *Sirt7*, *Slc1a5*, *Slc2a8*, *Slc7a1*, *Tlr1*, *Tlr2*, and *Tlr6* (Fig. 8C and D). Additionally, ferroptosis genes like *Slc7a11* and *Acsl4* were found to be suppressed by α-DIPA. These genes are involved in cystine/glutamate transport and lipid peroxidation, and their dysregulation has been implicated in the pathogenesis of neurodegenerative diseases. The α-DIPA regulation of these genes indicates a potential neuroprotective mechanism by preserving cellular redox balance and mitigating oxidative stress.

**Fig. 9:**
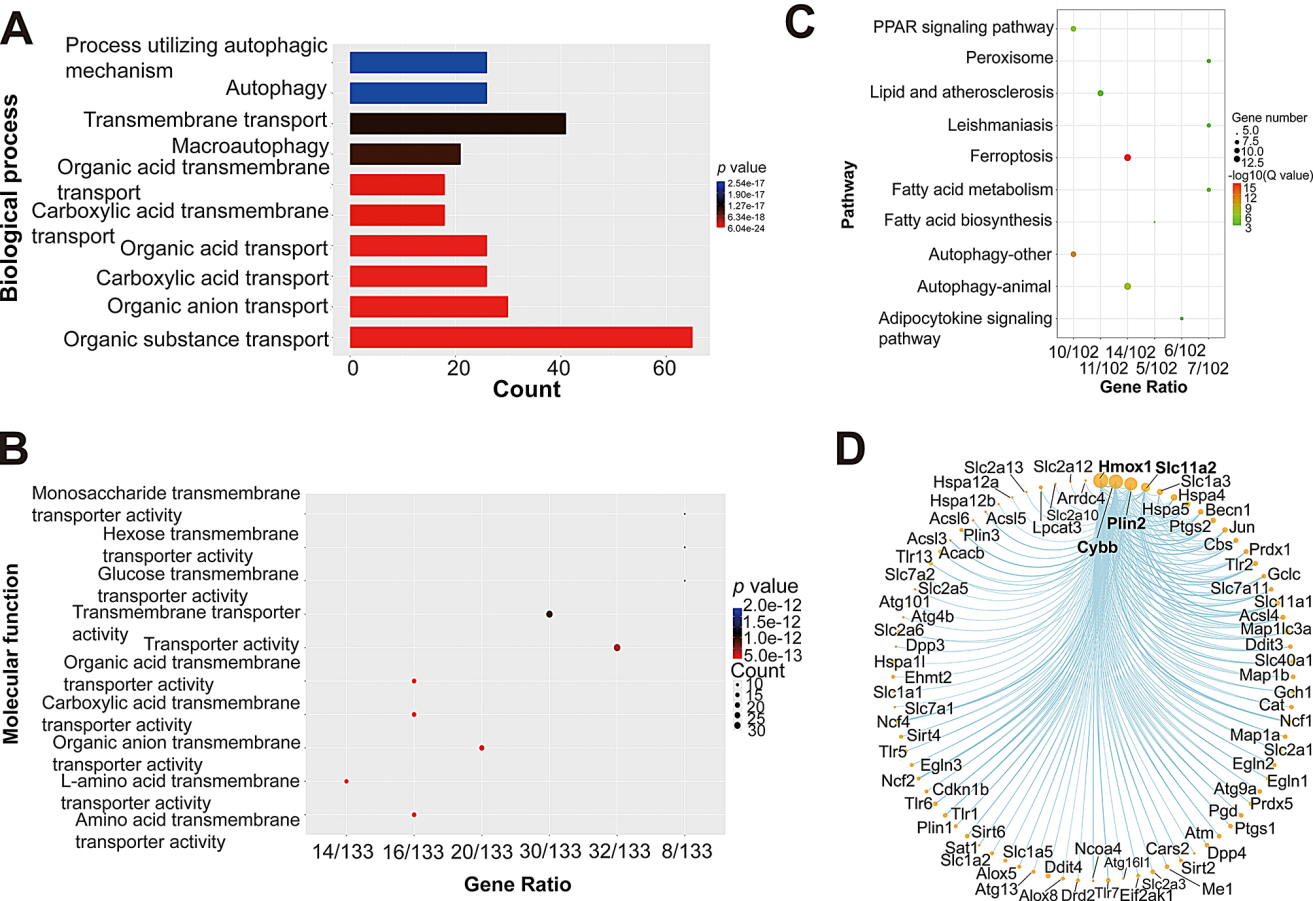
Gene ontology and pathway enrichment analysis of α-DIPA-regulated ferroptotic genes. (**A**) and (**B**) Bar graph and scatter plot showed the gene ontology of biological process (**A**) and molecular function (**B**) analysis results of α-DIPA-regulated 134 ferroptosis genes, respectively. (**C**) Scatter plot showed KEGG pathway enrichment analysis result of 134 ferroptosis-related genes regulated by α-DIPA. (**D**) Protein-protein interaction (PPI) analysis showed that ferroptosis genes such as *Hmox1* and *Slc11a2* interacted with multiple genes like *Cybb* and *Plin2* in α-DIPA-regulating networks. The online tool Search Tool for Recurring Instances of Neighbor (STRING) was used for PPI analysis

**Fig. 10:**
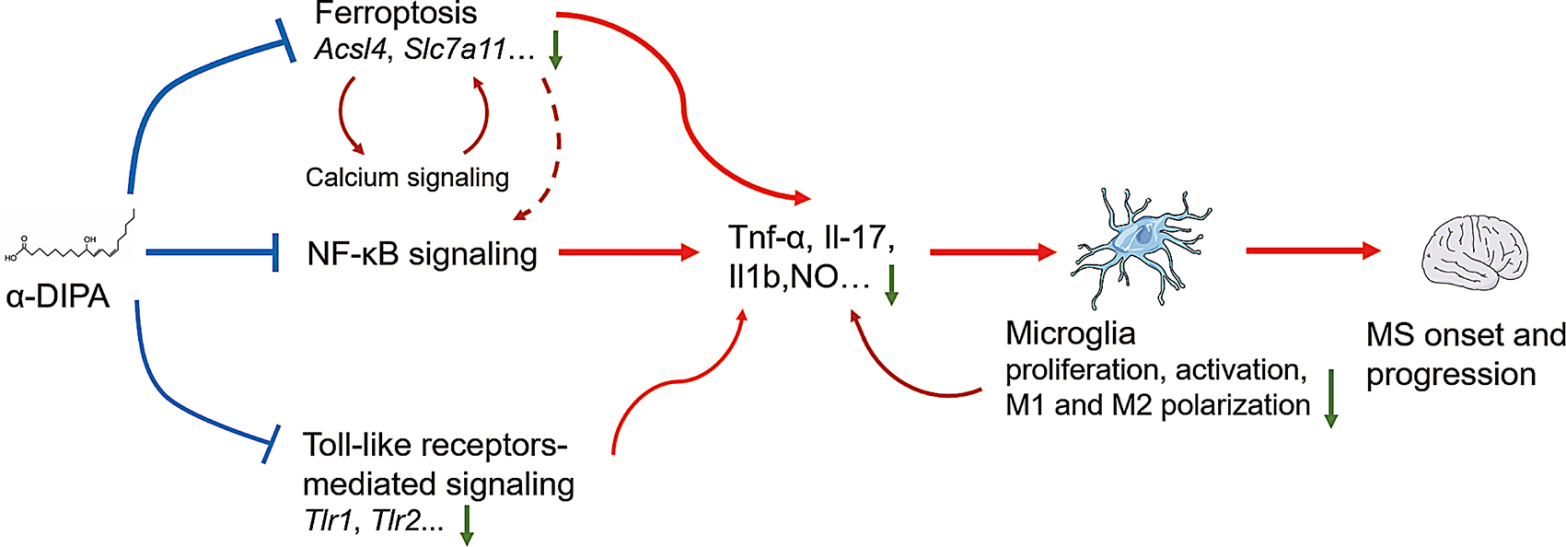
Schematic diagram of ferroptosis and NF-κB signaling involved in α-DIPA regulation of microglial inflammation and its potential role in MS pathology. Exogenous α-DIPA primarily suppresses microglial inflammation in vitro by regulating ferroptosis and NF-κB signaling, which inhibits cell proliferation, activation, M1 and M2 polarization, and NO release, thereby potentially impeding the onset and progression of MS driven by microglial inflammation. Moreover, α-DIPA may synergistically dampen microglial inflammatory responses by modulating calcium signaling and Toll-like receptor-mediated pathways

GO enrichment analysis showed the 134 genes involved in the biological processes of reactive oxygen species metabolic process, cellular response to stress, response to external stimulus, organic substance transport, amino acid transmembrane transport, and carboxylic acid transmembrane transport (Fig. 9A), and primary involved in regulating activity of several transmembrane transporter, including organic anion, carboxylic acid, organic acid, L-amino acid, hexose, and so on (Fig. 9B), which was similar to GO enrichment analysis results of Fig. 3D. In addition, KEGG pathway enrichment analysis showed that the 134 genes participated in ferroptosis, autophagy, PPAR signaling, peroxisome, fatty acid biosynthesis and metabolism, and adipocytokine signaling, etc. (Fig. 9C). PPI analysis showed those proteins tightly linked to each other, particularly, Hmox1, Cybb, Plin2, and Slc11a2 were the top four proteins that interacted most with other proteins such as Slc7a11, Acsl4, and Me1 (Fig. 9D). Taken together, these results suggested that α-DIPA likely inhibits microglial inflammation in vitro primarily through ferroptosis and NF-κB signaling, and possibly secondarily through calcium signaling and Toll-like receptor-mediated pathways (Fig. 10).

## Discussion

The CNS chronic inflammatory response mediated by glial cells, particularly microglia, underlies the etiopathology of MS. This autoimmune disease is characterized by demyelination and neurodegeneration in the CNS, where microglia play a pivotal role [55–64]. Upon activation, microglia undergo polarization into either a pro-inflammatory (M1) or anti-inflammatory (M2) state. The M1 state is typically associated with the secretion of pro-inflammatory cytokines such as TNF-α, IL-1β, and IL-6, which contribute to the inflammatory cascade, axonal demyelination, and subsequent neuronal cell death [65, 66]. Conversely, the M2 state supports tissue repair and the release of anti-inflammatory cytokines like IL-10 [67, 68]. The balance between these states is crucial, and dysregulation can lead to sustained inflammation and progressive neurodegeneration, marking microglial activation and polarization as critical early pathological events in MS [63, 69]. Despite advances in understanding the pathophysiology of MS, clinical treatment remains challenging. The condition is prone to relapses and is difficult to cure completely, with current therapeutic strategies primarily focusing on symptom management rather than addressing the root cause. Notably, novel therapeutic targets focusing on modulating microglial inflammatory responses hold promise. Intervening in the microglial activation process could potentially halt or even reverse disease progression, making it a fertile area for future research.

The role of immunometabolic regulation in the onset and progression of MS is significant. PUFAs like linoleic acid and their derivatives have been identified as key regulators of immune cell activity and CNS inflammation [28–30, 70–72]. These findings underscore the therapeutic potential of targeting metabolic pathways to modulate immune responses in MS. Previous studies have highlighted significant changes in the metabolic profiles of MS patients compared to HCs [49, 73–83]. Specifically, the plasma concentration of α-DIPA was markedly reduced in the plasma samples of MS patients [49], indicating a possible link between the changed level of α-DIPA and MS pathology. Additionally, elevated plasma levels of pro-inflammatory cytokines such as TNF-α and IL-17 and reduced levels of anti-inflammatory chemokines like IL-8 and MIP-1α have been observed in MS patients [49]. These findings suggest a complex interplay between metabolic networks and immune-inflammatory responses, including the negative relationships between α-DIPA with pro-inflammatory cytokines TNF-α and IL-17, further emphasizing the need to explore these interactions in the context of MS. However, the pathophysiological role of α-DIPA in microglial inflammation and MS pathogenesis remains unclear. The current study filled a significant gap in our understanding of the latent signaling pathways that mediate α-DIPA suppression of microglial inflammatory responses.

In this study, we found that exogenous α-DIPA treatment significantly blocked LPS-induced NO release from microglia cells in a concentration-dependent manner (*p* < 0.001) (Fig. 2). Furthermore, prior addition of 40 µM α-DIPA robustly inhibited LPS-induced microglia cells proliferation, activation, as well as M1 and M2 type polarization (Fig. 2). These results strongly suggested that endogenous α-DIPA might play a functional role in regulating microglia-evoked neuroinflammation in CNS, and that the deficiency of α-DIPA may be a pathological factor for MS onset and progression. In addition, RNA-Seq results showed that 40 µM α-DIPA treatment in microglia cells extensively and significantly inhibited mRNA expression levels of abundant proinflammatory genes, such as *Tnf*, *Ccl3*, *Ccl5*, *Bcl6*, and *Zfp36*, etc. (Fig. 4). Particularly, α-DIPA treatment largely repressed LPS-induced transcription levels of numerous genes involved in proinflammatory responses, TNF-α signaling, and IL-17 signaling (Figs. 4 and **5**). It’s worth noting that the circulating level of TNF-α and IL-17 was significantly increased in MS-affected patients relative to HCs [49], suggesting the absence of endogenous α-DIPA may be a causal factor resulting in activation of TNF-α and IL-17 in patients with MS. The suppression of IL-17 and TNF-α signaling by α-DIPA suggests a multifaceted approach to reducing inflammation. IL-17 and TNF-α are key drivers of pro-inflammatory responses, promoting the activation and recruitment of immune cells to sites of inflammation. By inhibiting these pathways, α-DIPA may reduce the release of additional pro-inflammatory cytokines and chemokines, thereby dampening the overall inflammatory response. This modulation could lead to a resolution of inflammation and a shift towards a more anti-inflammatory environment, which is crucial for the management of neuroinflammatory conditions like MS.

Even though a functional role of α-DIPA in regulating microglia inflammatory reactions was observed at cellular, molecular, and transcriptional level, however, it’s not clear which signaling pathways primarily mediate α-DIPA inhibition of microglial inflammation. KEGG pathway enrichment analysis showed the 778 overlapping DEGs significantly enriched in NF-κB signaling (Fig. 4B), and α-DIPA treatment greatly inhibited expression levels of LPS-induced multiple genes involved in NF-κB signaling (Fig. 6). The regulation of *Nfkb2* and *Cd40* by α-DIPA suggests that they could be potential targets for therapeutic intervention in MS. By targeting NF-κB signaling, α-DIPA may dampen the inflammatory cascade and promote a resolution of neuroinflammation. Notably, α-DIPA treatment also suppressed LPS-stimulated expression of numerous genes involved in ferroptosis pathway, including *Acsl4*, *Me1*, *Slc7a11*, *Hmox1*, and *Ptgs2* (Fig. 7C and D). More interestingly, we also observed the expression of multiple ferroptosis-related genes, such as *Acsl6*, *Alox5*, *Atg10*, *Slc1a2*, *Cars*, *Slc7a13*, *Dpp3*, *Ncf1*, *Ncoa6*, and *Tlr1*, were regulated specifically by α-DIPA but not LPS (Fig. 8). Previous studies have demonstrated that the expression level of *Acsl4* was significantly increased in mouse model of experimental autoimmune encephalomyelitis (EAE) and independent clinical cohorts [84–86], which suggesting ferroptosis signaling was likely activated in the inflammatory status in MS or animal model of EAE. Whereas, we observed the transcriptional levels of *Acsl4* and *Me1*, the key components in ferroptosis signaling, were robustly repressed by α-DIPA, indicating exogenous α-DIPA was able to suppress ferroptosis signaling by inhibiting *Acsl4* and *Me1* expression level. Moreover, the expression level of *arachidonate 5-lipoxygenase* (*Alox5*) was also regulated by α-DIPA. It’s well known that lipoxygenase (LOX) is responsible for catalyzing the oxidation of PUFAs on cell membrane to form lipid peroxides, which resulted in breakdown of cell membrane and cell death, eventually. These results implied that α-DIPA modulation of lipid peroxidation in ferroptosis signaling might be an important pathway to regulate microglia-evoked neuroinflammation. Furthermore, we also observed α-DIPA robustly inhibited the expression level of *Slc7a11*, which was synergistically transport extracellular cystine and cytoplasmic glutamic acid with Slc3a2, thereby to maintain physiological level of glutathione (GSH) and intracellular redox homeostasis. The result suggested that α-DIPA suppress microglia inflammatory responses might also mediated by regulation of intracellular redox homeostasis in ferroptosis signaling. In addition, it’s worth noting that α-DIPA-regulated DEGs mainly enriched in inorganic cation transmembrane transport, monoatomic ion transport and calcium signaling (Fig. 3D-F). Considering the tight interplay of calcium signaling with ferroptosis [87–90], our findings suggested that calcium signaling might play a synergistically regulatory role with ferroptosis in the process of α-DIPA inhibition of microglial inflammation. Interestingly, we also observed that α-DIPA extensively down-regulated the expressions of Toll-like receptors (Tlrs) members, including *Tlr1*, *Tlr2*, *Tlr6*, *Tlr7*, *Tlr9*, and *Tlr13* (Fig. 8C and D). It’s well known that Tlr family members involved in positive regulation of production of Tnf-α and IL-6 [91–94]. α-DIPA-attenuated Tlrs-mediated signaling probably synergistically down-regulate the expression level of Tnf-α and suppress Tnf-α-mediated proinflammatory responses. The integration of our findings revealed a complex interplay between α-DIPA effects on ferroptosis and NF-κB signaling in modulating microglial inflammation. α-DIPA regulation of ferroptosis-related genes, such as *Slc7a11* and *Acsl4*, suggests a reduction in cellular oxidative stress and lipid peroxidation, which are key drivers of ferroptotic cell death. By attenuating these processes, α-DIPA may reduce the cellular stress that would otherwise activate NF-κB signaling, a central pathway in the transcriptional control of inflammation. Furthermore, our data indicated that α-DIPA directly influences NF-κB signaling, as evidenced by the downregulation of *Nfkb2* and subsequent reduction in pro-inflammatory cytokines like Tnf-α and Il1b. This dual modulation of ferroptosis and NF-κB signaling by α-DIPA creates a feedback loop that potentially dampens microglial activation and the associated inflammatory response. In essence, the ability of α-DIPA to target both ferroptosis and NF-κB signaling pathways underscores its potential as a therapeutic agent in managing neuroinflammation associated with MS.

Our study focuses on α-DIPA’s ability to modulate microglial inflammation, which is a key aspect of MS pathology. Recent research has shown promise in remyelinating therapies, such as the work by Green and Chan at UCSF, which provided direct biologically validated imaging-based evidence of myelin repair induced by clemastine fumarate [95]. While clemastine fumarate has shown potential in myelin repair, our α-DIPA study offers a different approach by targeting inflammation, which is also crucial for disease progression. α-DIPA’s anti-inflammatory properties, as demonstrated in our transcriptomic analysis, suggest a complementary mechanism to therapies focusing on remyelination. Besides, we also compared α-DIPA effect with currently available disease-modifying drugs (DMTs) for MS. A systematic review and network meta-analysis of 11 DMTs showed that alemtuzumab was the most effective against annual relapse, while dimethyl fumarate and fingolimod were more effective for disability progression [96]. α-DIPA’s unique mechanism, targeting ferroptosis and inflammation, differentiates it from these DMTs and could potentially offer an alternative or adjunct therapy, especially considering its effects on neuroinflammation. In addition, the landscape of progressive MS treatments is evolving, with several therapies under investigation, including sphingosine receptor modulators (such as siponimod), tyrosine kinase inhibitors (such as masitinib), and monoclonal antibodies (for example, natalizumab and ocrelizumab) [97]. Our study contributes to this field by providing insights into the α-DIPA modulation of neuroinflammation, which is a common pathogenic process in both relapsing-remitting and progressive MS. The potential of α-DIPA to influence key inflammatory pathways may provide a novel therapeutic strategy that could be applicable across different MS subtypes. Furthermore, compared to the emerging treatment options such as mesenchymal stem cell therapy, which are being explored for their regenerative and neuroprotective properties for neurological disorders like MS [98]. α-DIPA, with its anti-inflammatory and potential neuroprotective effects, may fit into this category of novel treatments, offering a small molecule approach to target inflammation in MS. In contrast to conventional anti-inflammatory drugs that may have limited effects on the progressive course of MS, our study introduces a novel perspective on the treatment of neuroinflammation associated with MS by revealing α-DIPA as a regulator of ferroptosis, a form of cell death distinct from apoptosis or necrosis. Unlike traditional therapeutic targets that focus on inhibiting specific inflammatory cytokines or enzymes, α-DIPA’s mechanism of action targets the underlying metabolic pathway of ferroptosis, which has been increasingly recognized for its role in neurodegenerative diseases. By modulating the ferroptosis pathway, α-DIPA potentially addresses a critical unmet need in MS treatment: the mitigation of neuroinflammation through the regulation of glial cell death mechanisms. This distinct regulatory effect on ferroptosis positions α-DIPA as a promising candidate for further investigation in the development of novel therapeutics for MS.

However, there are some limitations in the current study. First, the sample size of MS patients and HCs, though statistically adequate based on our power analysis, is modest and may limit the generalizability of our findings. Larger, multicenter studies with more diverse cohorts are needed to confirm our results and explore potential subgroup effects. Besides, we recognize that potential confounders such as lifestyle factors, including diet and physical activity, could influence α-DIPA levels and subsequent biological effects. In this study, we did not systematically collect data on these factors, which could have introduced variability into our results. Future studies should incorporate comprehensive lifestyle assessments to control for these potential confounders and provide a more nuanced understanding of α-DIPA’s role in MS pathology. We also acknowledge that our study’s cross-sectional design limits our ability to establish causality between α-DIPA levels and MS disease activity. Longitudinal studies are warranted to further explore the temporal relationship and potential causal mechanisms. Second, our data showed that exogenous α-DIPA treatment was capable of inhibiting LPS-induced microglia inflammatory responses, nevertheless, the biological function of α-DIPA in regulating microglia inflammation was only explored in vitro, more strong and direct evidences collected from in vivo assays and animal model of MS were currently absent from this study. The use of BV-2 cells, an immortalized microglial cell line, may not fully replicate the complex behavior of primary microglia in vivo. Although BV-2 cells are widely used as a model for studying microglial function, they may exhibit differences in gene expression and response to extrinsic stimuli compared to primary microglia. This limitation suggests that our findings should be interpreted with caution when considering their applicability to the native microglial environment in the CNS. Therefore, further investigations including neurobehavioral assessments and histopathological evaluation of α-DIPA-treated EAE model, molecular and cellular analysis of α-DIPA-responsive inflammatory markers, as well as pharmacokinetic and pharmacodynamic testing of α-DIPA, are needed to be performed to investigate the physiological and pharmacological role of α-DIPA in regulating microglia-elicited neuroinflammation in CNS, and *bona fide* role of endogenous α-DIPA in influencing MS course. Third, transcriptomic analysis has demonstrated that exogenous α-DIPA treatment extensively and significantly inhibited mRNA expression level of numerous genes involved in regulation of inflammatory responses, such as TNF-α and IL-17 signaling, as well as NF-κB signaling. Similarly, our previous research found that concentration of TNF-α and IL-17 was significantly increased in patients with MS [49, 50]. However, little is known about whether the deficiency of α-DIPA in MS-affected patients directly enhance TNF-α and IL-17 signaling and microglia proinflammatory reactions in the CNS, therefore, validation study is needed to be carried out to demonstrate that decreased plasma level of endogenous α-DIPA indeed facilitates inflammation in peripheral system and even in the CNS. Fourth, transcriptomic analysis showed that exogenous α-DIPA treatment vigorously inhibited the expression levels of abundant ferroptosis-related genes in microglia, such as *Acsl4*, *Slc7a11*, *Me1*, and *Cars*. Although it’s well known that ferroptosis is tightly related to neuroinflammation-evoked diseases, such as MS and Alzheimer’s disease, however, it remains elusive about whether endogenous α-DIPA is capable of regulating microglial inflammation in the CNS mainly through ferroptosis pathway. Thus, further investigation is essential to be conducted to determine the role of ferroptosis involved in α-DIPA inhibition of microglia inflammation and α-DIPA-regulating MS pathogenesis.

## Conclusion

In this study, we demonstrated that exogenous α-DIPA treatment effectively inhibits LPS-induced microglial inflammation, including microglial proliferation, activation, and polarization, as well as the release of NO and pro-inflammatory factors such as TNF-α. Transcriptomic analysis further revealed that α-DIPA robustly suppresses inflammatory signaling pathways, including IL-17 and TNF-α signaling, potentially via its modulation of ferroptosis and NF-κB pathways. These findings deepen our understanding of the molecular mechanisms underpinning α-DIPA’s anti-inflammatory effects and identify it as a promising therapeutic target for microglial-driven neuroinflammatory conditions.

## Electronic supplementary material

Below is the link to the electronic supplementary material.

Supplementary Material 1: Figure S1. Volcano plot shows differentially expressed genes regulated by LPS in BV-2 cells. A total of 2,138 differentially expressed genes, including 1,416 up-regulated (such as *Tnf*) and 722 down-regulated genes (such as *Ccnb2*), were identified in LPS treatment group compared to control, respectively. Log_2_(FC) > 1.0 or Log_2_(FC) < -1.0, and *p* < 0.05 was used to identify up-regulated or down-regulated genes, respectively. Three biological replicates were used in each group.

Supplementary Material 2: Figure S2. Volcano plot shows ferroptosis-related genes regulated by LPS in BV-2 cells. A total of 27 differentially expressed genes related to ferroptosis, including 23 up-regulated (such as *Slc7a11*) and 4 down-regulated genes (such as *Ncoa4*), were identified in LPS treatment group compared to control, respectively. Log_2_(FC) > 1.0 or Log_2_(FC) < -1.0, and *p* < 0.05 was used to identify up-regulated or down-regulated ferroptosis genes, respectively. Three biological replicates were used in each group.

Supplementary Material 3

## Data Availability

The original contributions presented in the study are included in the article/Supplementary Material. Further inquiries can be directed to the corresponding authors.

## Abbreviations

Alox: Arachidonate lipoxygenase
α-DIPA: α-dimorphecolic acid
BrdU: 5-bromo-2’-deoxyuridine
CNS: Central nervous system
DAM: Differentially abundant metabolite
DEG: Differentially expressed gene
EAE: Experimental autoimmune encephalomyelitis
ELISA: Enzyme-linked immunosorbent assay
FACS: Fluorescence-activated cell sorting
GO: Gene ontology
GSH: Glutathione
HCs: Healthy controls
HODE: Hydroxyoctadecaenoic acid
KEGG: Kyoto encyclopedia of genes and genomes
LA: Linoleic acid
LC-MS/MS: Liquid chromatography-mass spectrometry/ mass spectrometry
LOX: Lipoxygenase
LPS: Lipopolysaccharide
L-NMMA: NG-monomethyl-L-arginine
MS: Multiple sclerosis
NO: Nitric oxide
OPLS-DA: Orthogonal partial least squares-discriminant analysis
PCA: Principal component analysis
PLS-DA: Partial least squares-discriminant analysis
PPI: Protein-protein interaction
PUFA: Polyunsaturated fatty acid
Tlr: Toll-like receptor
