# Supplementary figures and images for: Transcriptomic profiling identifies ferroptosis and NF-κB signaling involved in α-dimorphecolic acid regulation of microglial inflammation
Source: J Transl Med. 2025 Mar 4;23:260. doi: 10.1186/s12967-025-06296-7 (PMC11877847; doi:10.1186/s12967-025-06296-7)

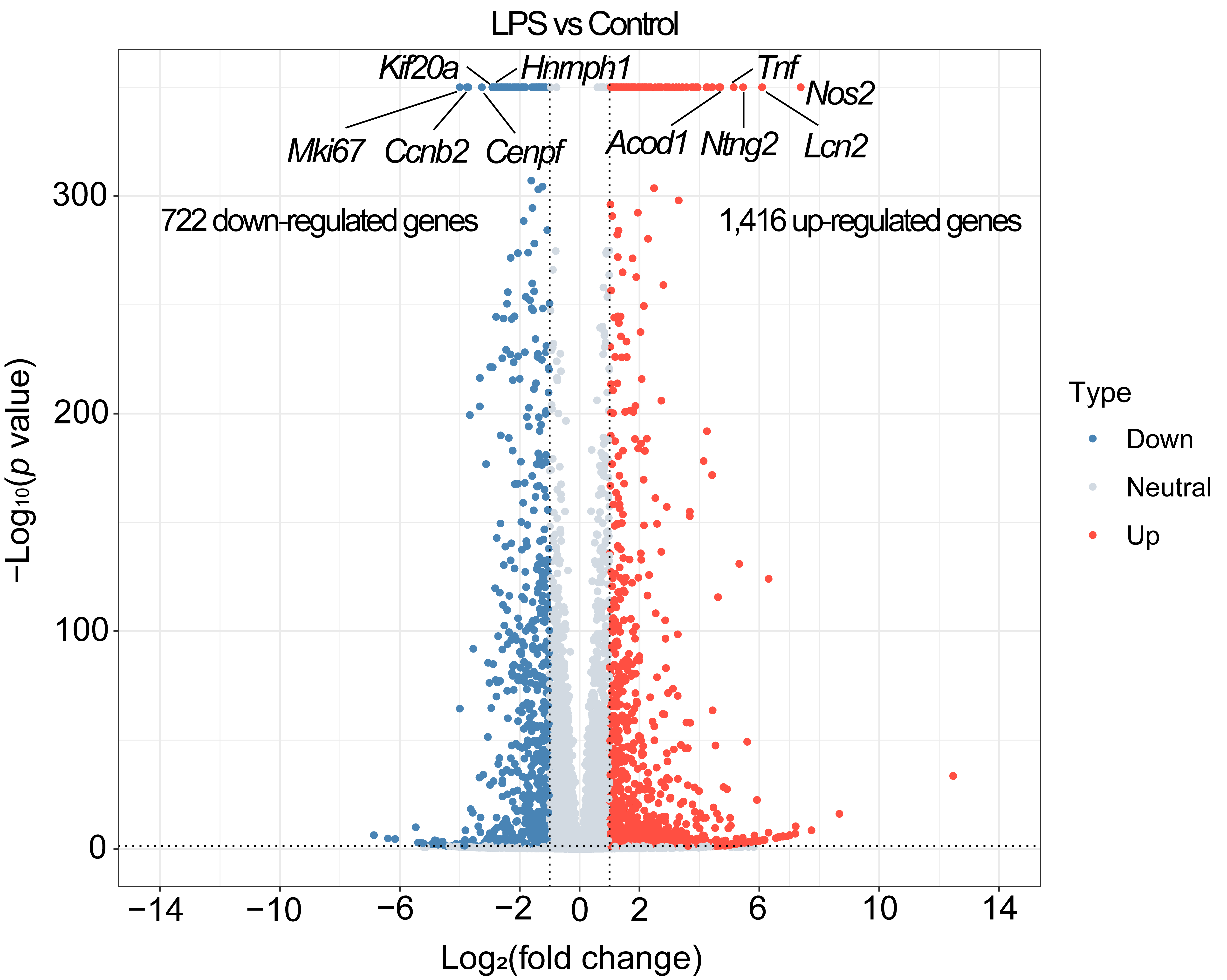

Supplement: Supplementary file 1 — Supplementary Material 1: Figure S1. Volcano plot shows differentially expressed genes regulated by LPS in BV-2 cells. A total of 2,138 differentially expressed genes, including 1,416 up-regulated (such as Tnf) and 722 down-regulated genes (such as Ccnb2), were identified in LPS treatment group compared to control, respectively. Log2(FC) > 1.0 or Log2(FC) < -1.0, and p < 0.05 was used to identify up-regulated or down-regulated genes, respectively. Three biological replicates were used in each group. [file 12967_2025_6296_MOESM1_ESM.tif]

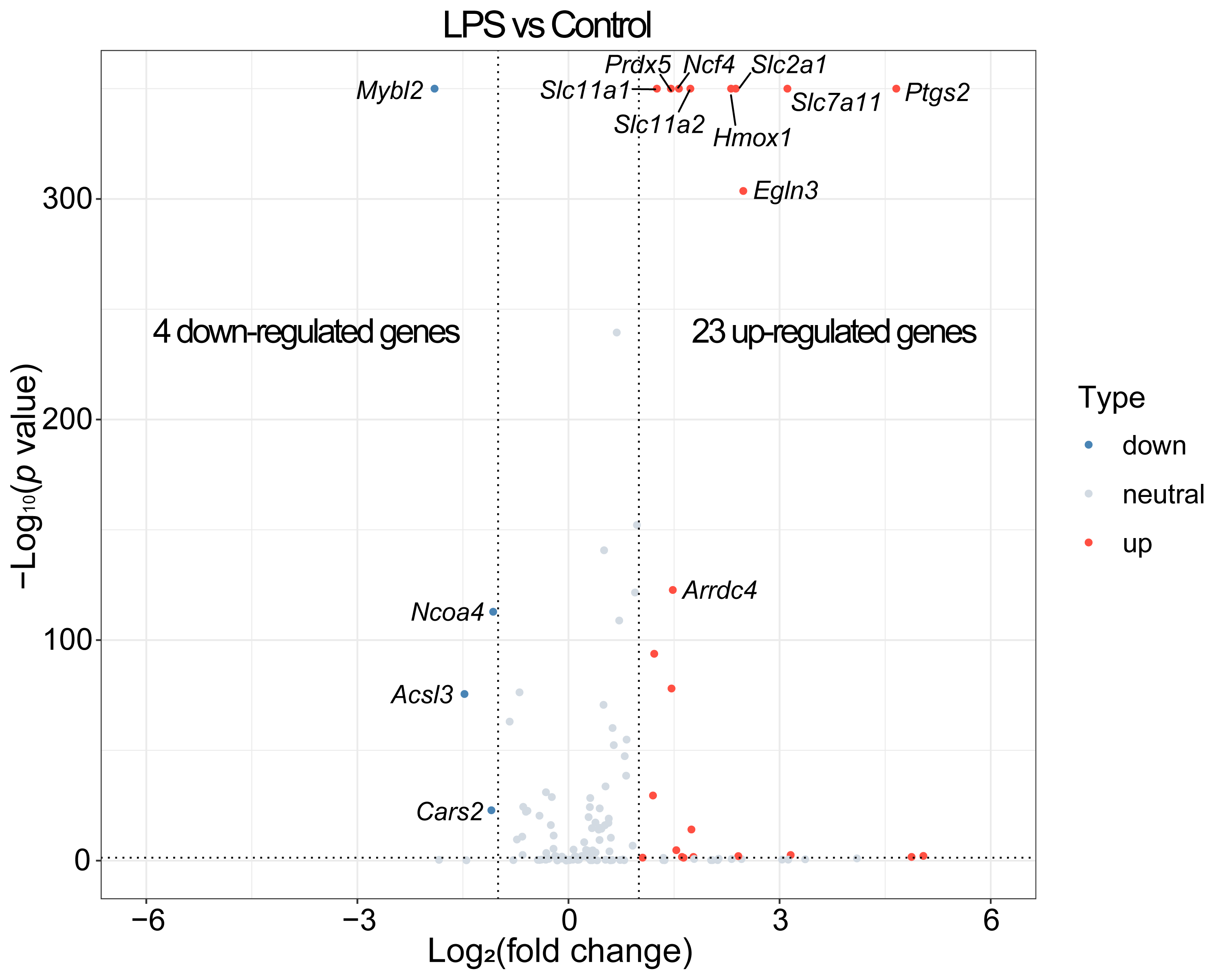

Supplement: Supplementary file 2 — Supplementary Material 2: Figure S2. Volcano plot shows ferroptosis-related genes regulated by LPS in BV-2 cells. A total of 27 differentially expressed genes related to ferroptosis, including 23 up-regulated (such as Slc7a11) and 4 down-regulated genes (such as Ncoa4), were identified in LPS treatment group compared to control, respectively. Log2(FC) > 1.0 or Log2(FC) < -1.0, and p < 0.05 was used to identify up-regulated or down-regulated ferroptosis genes, respectively. Three biological replicates were used in each group. [file 12967_2025_6296_MOESM2_ESM.tif]
