# Supplementary material for: Transcriptomic profiling identifies ferroptosis and NF-κB signaling involved in α-dimorphecolic acid regulation of microglial inflammation
Source: J Transl Med. 2025 Mar 4;23:260. doi: 10.1186/s12967-025-06296-7 (PMC11877847; doi:10.1186/s12967-025-06296-7)
Supplement: Supplementary file 3 — Supplementary Material 3 [file 12967_2025_6296_MOESM3_ESM.docx]

**Supplementary table**

**Table S1** The demographic information of patients with MS and healthy controls.

| **Characteristics** | **Patients with MS** | **HCs** | ***P* value** |
| --- | --- | --- | --- |
| Number | 22 | 21 | NA |
| Gender, male/female | 8/14 | 8/13 | NA |
| Age (y), mean ± SD. | 34.8 ± 7.5 | 33.3 ± 8.5 | NS |
| BMI (kg/m^2^), mean ± SD. | 21.6 ± 2.6 | 22.1 ± 3.6 | NS |
| MS duration (y), mean ± SD. | 7.4 ± 5.0 | NA |  |
| Hypertension | 0 | 0 |  |
| Hyperlipidemia | 0 | 0 |  |
| Diabetes mellitus | 0 | 0 |  |
| Autoimmune conditions | 0 | 0 |  |
| Active infections | 0 | 0 |  |
| Treatment of immune-modulating drugs within 18 months | 0 | 0 |  |

HCs, healthy controls; MS, multiple sclerosis; BMI, body mass index; SD, standard deviation; NA, not available; NS, not significant.
